# Supplementary material for: Vibration threshold in non-diabetic subjects
Source: PLoS One. 2020 Oct 7;15(10):e0237733. doi: 10.1371/journal.pone.0237733 (PMC7540842; doi:10.1371/journal.pone.0237733)

$$VPT_{bigtoe} = -90.09 + 0.24 * AGE + 0.50 * HEIGHT$$

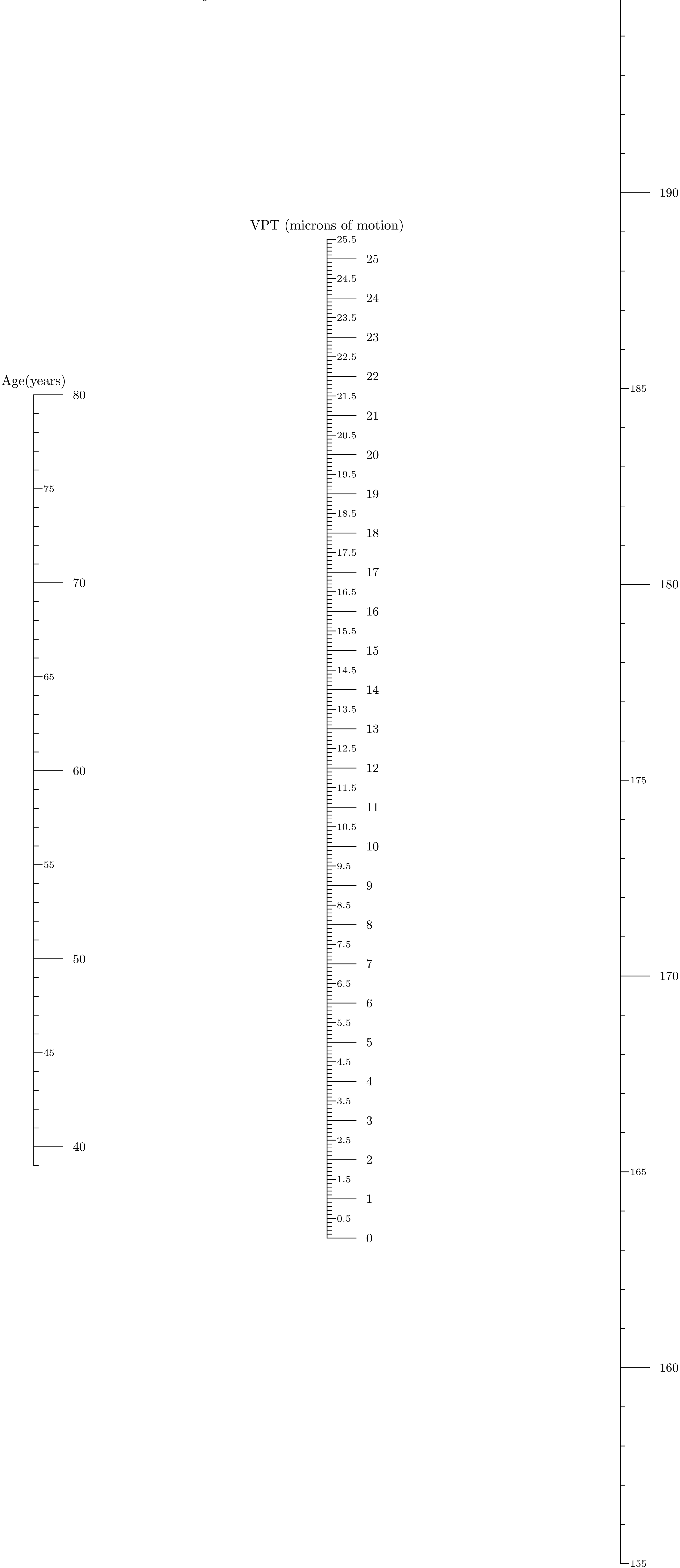

$$VPT_{instep} = -98.70 + 0.35 * AGE + 0.52 * HEIGHT$$

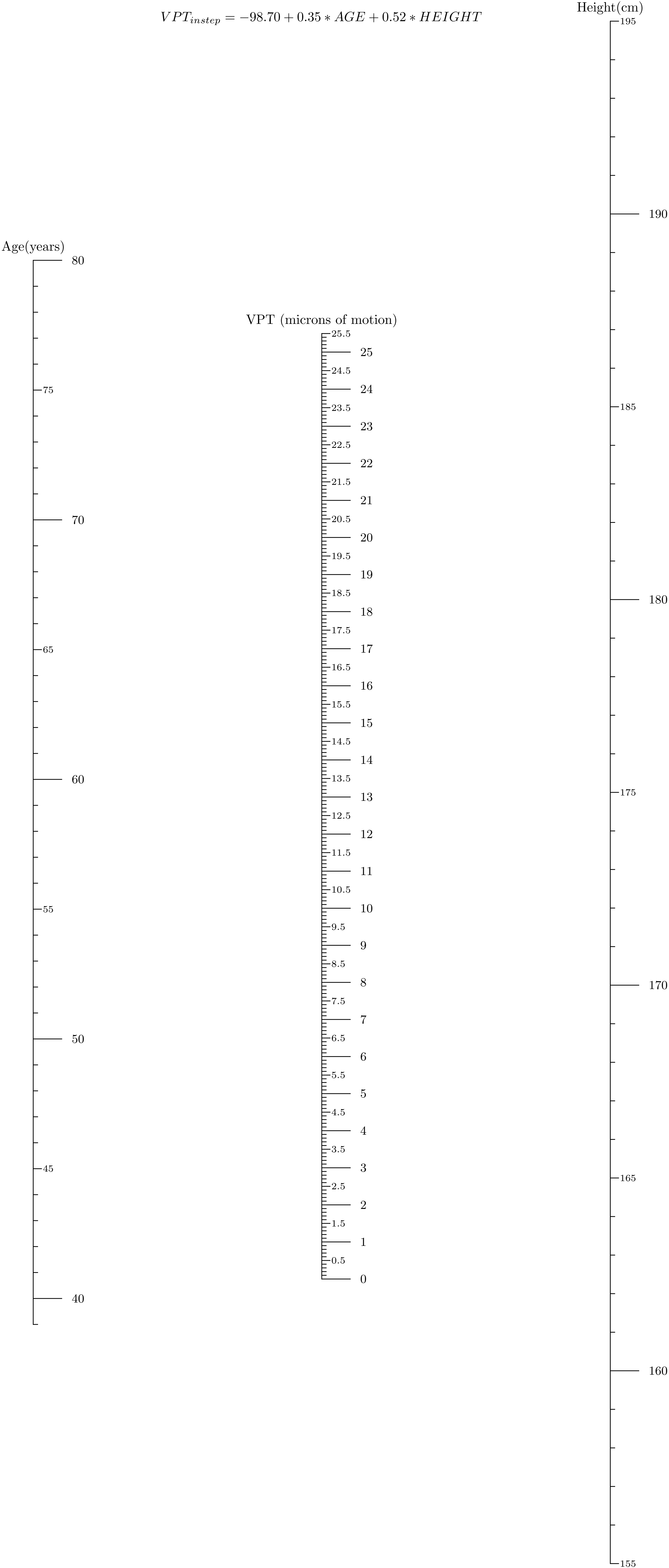

$$VPT_{latmalleolus} = -70.27 + 0.26 * AGE + 0.37 * HEIGHT$$

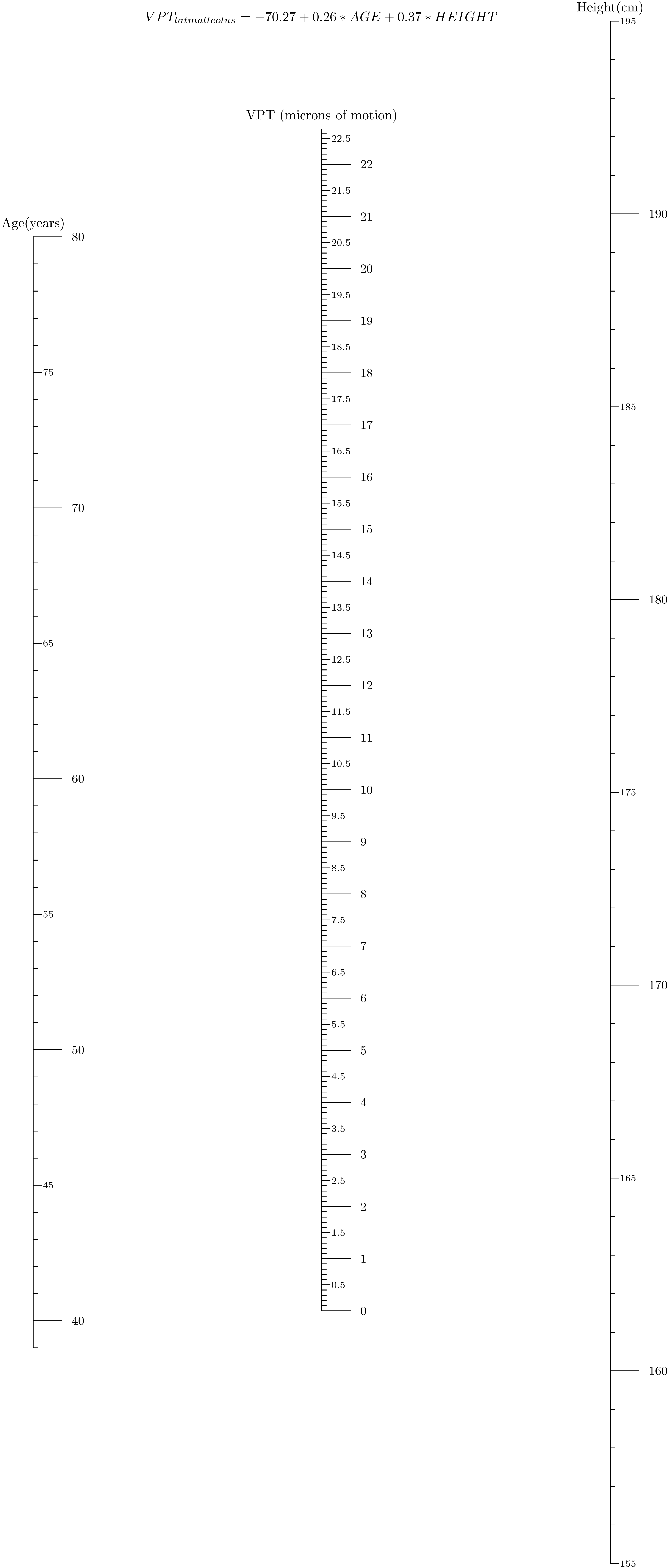

$$VPT_{wrist} = -4.55 + 0.06 * AGE + 0.02 * HEIGHT$$

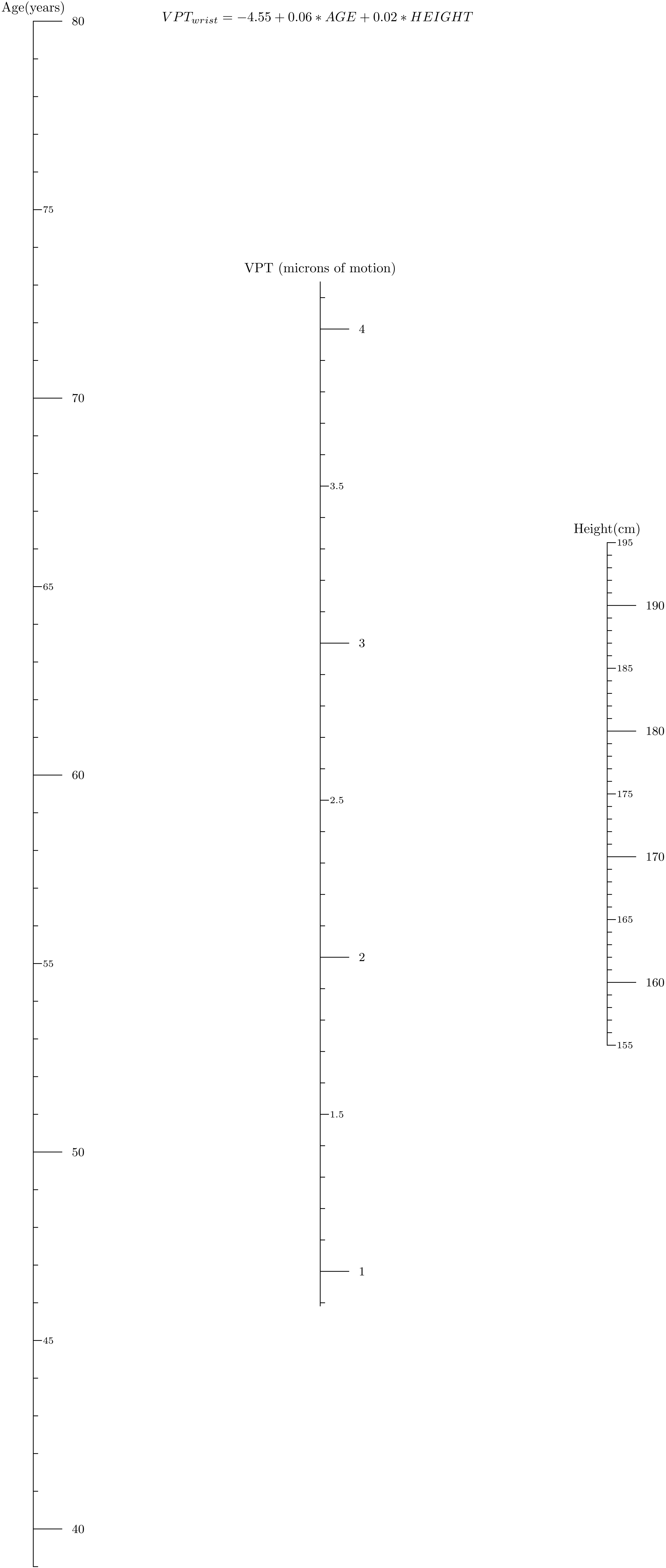

$$VPT_{latmalleolus} = -70.27 + 0.26 * AGE + 0.37 * HEIGHT$$

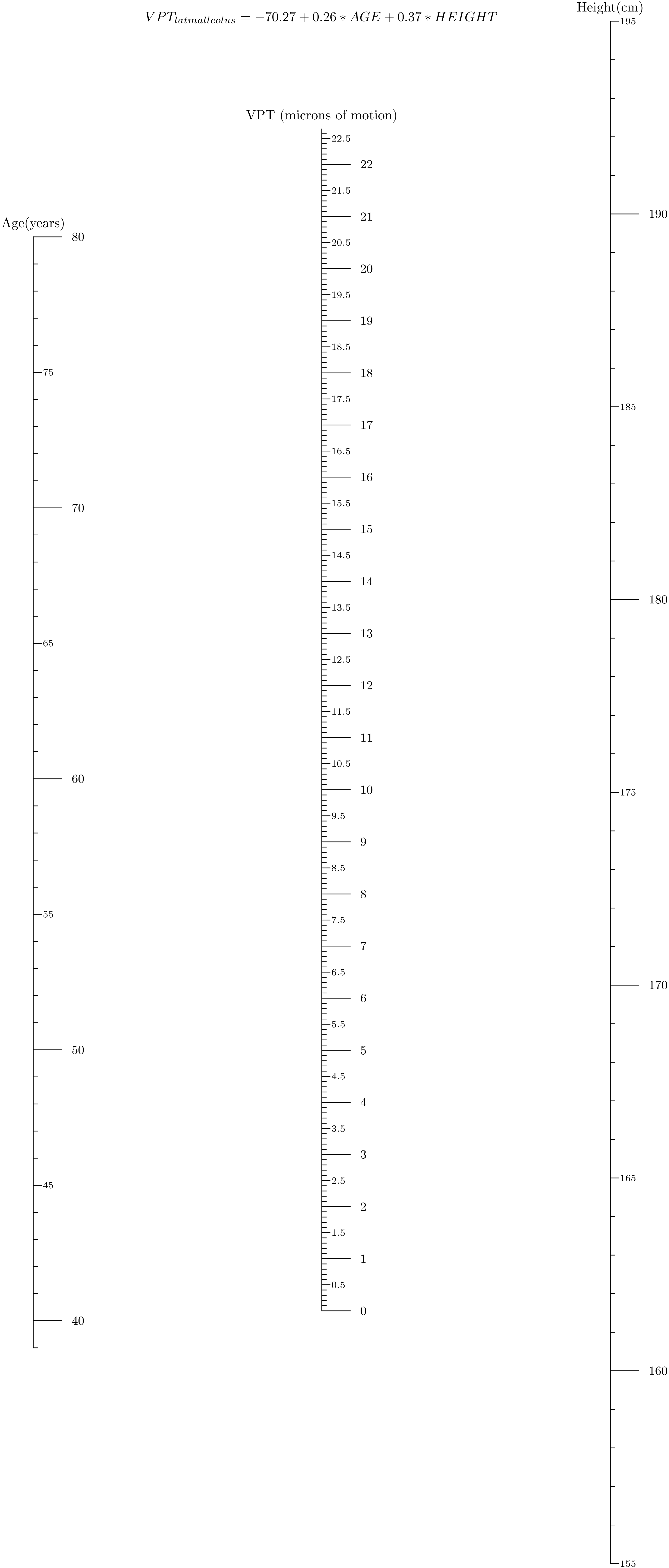

Supplement: S1 Fig — By filling in age and height of the patient on the outer scales, connecting the two points by drawing a straight line, the normal value for the VPT (vibration perception threshold) can be read from the middle scale. (PDF) [file pone.0237733.s001.pdf]
