# Supplementary material for: Vibration threshold in non-diabetic subjects
Source: PLoS One. 2020 Oct 7;15(10):e0237733. doi: 10.1371/journal.pone.0237733 (PMC7540842; doi:10.1371/journal.pone.0237733)
Supplement: S1 File — Patients who underwent a third measurement were excluded from the study population and the statistical analyses were carried out as described in the Methodology section. (DOCX) [file pone.0237733.s002.docx]

**S2. Sensitivity analysis.**

Patients who underwent a third measurement were excluded from the study population and the mean between the two included measurements of VPT was calculated. The clinical characteristics are presented in Table S2.1. Table S2.2 provides a comparison of clinical characteristics of the group without a third measurement and the group with a third measurement. Results of the univariate linear regression analyses are displayed in Table S2.3. Age and height were identified as independent determinants of VPT at the big toes (Table S2.4). The results at the other points of measurement are displayed in Table S2.4. The distribution of subjects dependent on age and height and the measured VPT is displayed in Table S2.5. The corresponding regression coefficients according to the 95th percentile were calculated and are shown in Table S2.6. The model can be summarized with the following corresponding regression equations:

$$\Upsilon_{VPT\_BIGTOE}= -79.76+ 0.18\times AGE + 0.45\times HEIGHT$$

$$\Upsilon_{VPT\_INSTEP}= -88.53+0.24\times AGE+0.49\times HEIGHT$$

$$\Upsilon_{VPT\_LATMALLEOLUS}= -63.35+0.23\times AGE+0.34\times HEIGHT$$

$$\Upsilon_{VPT\_WRIST}= -7.33+0.03\times AGE+0.04\times HEIGHT$$

**Table S2.1. Clinical characteristics of the cohort.**

| **Variable** |  | **Range** |
| --- | --- | --- |
| Number | 120 |  |
| Age, years | 57.5 [48;64] | 21-77 |
| Sex, n(%) male | 58 (48.3) |  |
| Height, cm | 173.81 ± 9.06 | 152.50-195.50 |
| Weight, kg | 80.78 ± 14.66 | 54.00-118.80 |
| BMI, kg/m^2^ | 26.63 ± 3.73 | 18.90-35.00 |
| Systolic blood pressure, mmHg | 125.73 ± 16.09 | 93.00-175.00 |
| Diastolic blood pressure, mmHg | 74.35 ± 11.57 | 50.00-100.00 |
| Hypertension, n(%) | 26 (21.7) |  |
| History of cardiovascular disease, n(%) | 9 (7.7) |  |
| Medication use, n(%) | 61 (51.3) |  |
| Statins, n(%) | 13 (10.8) |  |
| Antiretroviral medication, n(%) | 0 (0) |  |
| Smoking, n(%) | 16 (13.3) |  |
| Alcohol intake, units/day | 0 [0;0.70] | 0-2.58 |
| *Relevant blood values* |  |  |
| Blood glucose, mmol/L | 5.39 ± 0.55 | 3.70-6.80 |
| HbA1c, mmol/mol | 37.00 [34.25;39.00] | 30.00-47.00 |
| Vitamin B12, pmol/L | 308.0 [232.0;373.5] | 146.0-750.0 |
| Folic acid, nmol/L | 15.1 [11.0;21.3] | 3.2-45.4 |

**Table S2.2. Comparison**

| **Variable** | **No 3^rd^ measurement** | **3^rd^ measurement** | **P-value** |
| --- | --- | --- | --- |
| Number | 120 | 85 |  |
| Age, years | 57.5 [48;64] | 61.0 [55;66] | 0.01 |
| Sex, n(%) male | 58 (48.3) | 50 (58.8) | 0.14 |
| Height, cm | 173.81 ± 9.06 | 175.35 ± 9.36 | 0.24 |
| Weight, kg | 80.78 ± 14.66 | 85.99 ± 14.64 | 0.01 |
| BMI, kg/m^2^ | 26.63 ± 3.73 | 27.97 ± 4.26 | 0.02 |
| Systolic blood pressure, mmHg | 125.73 ± 16.09 | 129.65 ± 13.14 | 0.07 |
| Diastolic blood pressure, mmHg | 74.35 ± 11.57 | 75.70 ± 9.94 | 0.38 |
| Hypertension, n(%) | 26 (21.7) | 30 (36.1) | 0.03 |
| History of cardiovascular disease, n(%) | 9 (7.7) | 6 (7.2) | 0.90 |
| Medication use, n(%) | 61 (51.3) | 51 (60.7) | 0.18 |
| Statins, n(%) | 13 (10.8) | 7 (8.2) | 0.54 |
| Antiretroviral medication, n(%) | 0 (0) | 0 (0) | 1.00 |
| Smoking, n(%) | 16 (13.3) | 12 (14.1) | 0.87 |
| Alcohol intake, units/day | 0 [0;0.70] | 0.13 [0;0.88] | 0.10 |
| *Relevant blood values* |  |  |  |
| Blood glucose, mmol/L | 5.39 ± 0.55 | 5.42 ± 0.53 | 0.73 |
| HbA1c, mmol/mol | 37.00 [34.25;39.00] | 37.00 [35.00;39.00] | 0.37 |
| Vitamin B12, pmol/L | 308.0 [232.0;373.5] | 294.0 [234.0;359.5] | 0.69 |
| Folic acid, nmol/L | 15.1 [11.0;21.3] | 15.3 [13.3;22.9] | 0.24 |

**Table S2.3. Univariate linear regression analyses of determinants of VPT.**

| **Measurement location** | **Independent variable** | **B (95% CI)** | **Standardized *β*** | **P-value** |
| --- | --- | --- | --- | --- |
| **Big toe** | Age, years | 0.02 (0.01-0.03) | 0.42 | <0.001 |
|  | Height, cm | 0.02 (0.01-0.03) | 0.38 | <0.001 |
|  | Weight, kg | 0.01 (0.01-0.02) | 0.36 | <0.001 |
|  | Sex | -0.30 (-0.47--0.12) | -0.30 | 0.001 |
|  | Smoking behavior | -0.09 (-0.35-0.18) | -0.06 | 0.52 |
| **Instep** | Age, years | 0.02 (0.01-0.03) | 0.40 | <0.001 |
|  | Height, cm | 0.02 (0.01-0.03) | 0.35 | <0.001 |
|  | Weight, kg | 0.01 (0.01-0.02) | 0.38 | <0.001 |
|  | Sex | -0.40 (-0.58--0.22) | -0.38 | <0.001 |
|  | Smoking behavior | -0.05 (-0.33-0.24) | -0.03 | .73 |
| **Lateral malleolus** | Age, years | 0.02 (0.01-0.02) | 0.42 | <0.001 |
|  | Height, cm | 0.02 (0.01-0.03) | 0.32 | <0.001 |
|  | Weight, kg | 0.01 (0.01-0.02) | 0.37 | <0.001 |
|  | Sex | -0.36 (-0.51--0.20) | -0.39 | <0.001 |
|  | Smoking behavior | -0.09 (-0.33-0.15) | -0.07 | 0.47 |
| **Wrist** | Age, years | 0.01 (0.001-0.01) | 0.22 | 0.02 |
|  | Height, cm | 0.01 (0-0.01) | 0.17 | 0.06 |
|  | Weight, kg | 0.01 (0.001-0.01) | 0.24 | 0.01 |
|  | Sex | -0.22 (-0.34--0.10) | -0.31 | <0.001 |
|  | Smoking behavior | -0.003 (-0.19-0.19) | -0.003 | 0.97 |

CI: confidence interval

**Table S2.4. Forward selection multiple linear regression models of determinants of VPT^1^.**

| **Measurement location** | **Independent variable** | **B (95% CI)** | **Standardized *β*** | **P-value** |
| --- | --- | --- | --- | --- |
| **Big toe** | Age, years | 0.03 (0.02-0.03) | 0.55 | <0.001 |
|  | Height, cm | 0.03 (0.02-0.04) | 0.51 | <0.001 |
| **Instep** | Age, years | 0.02 (0.02-0.03) | 0.49 | <0.001 |
|  | Height, cm | 0.02 (0.01-0.03) | 0.34 | 0.001 |
|  | Weight, kg | 0.01 (0-0.01) | 0.19 | 0.05 |
| **Lateral malleolus** | Age, years | 0.02 (0.02-0.03) | 0.51 | <0.001 |
|  | Height, cm | 0.01 (0-0.03) | 0.28 | 0.01 |
|  | Sex | -0.21 (-0.39--0.03) | -0.23 | 0.03 |
| **Wrist** | Age, years | 0.01 (0-0.01) | 0.24 | 0.01 |
|  | Sex | -0.23 (-0.35--0.11) | -0.33 | <0.001 |

^1^ Forward selection, P-value to enter ≤ 0.05

CI: confidence interval

**Table S2.5. Distribution of subjects and measured VPT dependent on age and height grouping.**

| **Age, years** | **Number of subjects** | **Big toes^1^** | **Insteps^1^** | **Lateral malleoli^1^** | **Wrists^1^** |
| --- | --- | --- | --- | --- | --- |
| ≤ 39 | 8 | 1.06 (-0.004-2.13) | 1.32 (0.05-2.60) | 1.12 (0.24-2.00) | 0.44 (0.20-0.68) |
| 40-49 | 30 | 2.52 (0.84-4.19) | 2.31 (0.95-3.67) | 2.27 (1.20-3.34) | 0.59 (0.46-0.72) |
| 50-59 | 32 | 2.61 (1.78-3.46) | 3.59 (1.90-5.27) | 3.04 (1.89-4.19) | 0.72 (0.52-0.91) |
| 60-69 | 38 | 2.15 (1.29-3.01) | 2.57 (1.64-3.49) | 2.44 (1.79-3.09) | 0.62 (0.39-0.84) |
| 70-80 | 12 | 5.22 (2.84-7.60) | 6.13 (3.36-8.89) | 4.73 (2.83-6.62) | 0.84 (0.49-1.18) |

^1^ mean VPT (95% CI)

| **Height, cm** | **Number of subjects** | **Big toes^1^** | **Insteps^1^** | **Lateral malleoli^1^** | **Wrists^1^** |
| --- | --- | --- | --- | --- | --- |
| <160 | 5 | 0.80 (0.45-1.16) | 1.09 (0.77-1.41) | 1.19 (0.70-1.68) | 0.51 (0.31-0.72) |
| 160-169 | 35 | 1.95 (1.28-2.61) | 2.15 (1.30-3.00) | 2.07 (1.37-2.80) | 0.57 (0.43-0.71) |
| 170-179 | 49 | 2.61 (1.81-3.41) | 3.31 (2.22-4.39) | 3.02 (2.24-3.79) | 0.83 (0.60-1.06) |
| 180-190 | 24 | 3.97 (2.23-5.71) | 4.48 (2.53-6.42) | 3.89 (2.74-5.05) | 0.76 (0.56-0.95) |
| >190 | 7 | 8.71 (1.41-16.00) | 7.25 (0.12-14.39) | 6.11 (0.33-11.89) | 0.71 (0.30-1.13) |

^1^ mean VPT (95% CI)

**Table S2.6. Quantile regression.**

|  | **Variable** | **Original model ^1^** | | | | **Model after bootstrap resampling ^2^** | | | |
| --- | --- | --- | --- | --- | --- | --- | --- | --- | --- |
|  |  | **RC** | **SE** | **95% CI** | **P-value** | **RC** | **SE** | **95% CI** | **P-value** |
| **Big toe** | Constant | -79.76 | 30.51 | -140.17--19.34 | 0.010 | -79.76 | 16.31 | -112.06--47.45 | <0.001 |
|  | Age, years | 0.18 | 0.13 | -0.08-0.44 | 0.177 | 0.18 | 0.08 | 0.03-0.34 | 0.023 |
|  | Height, cm | 0.45 | 0.16 | 0.13-0.77 | 0.006 | 0.45 | 0.11 | 0.23-0.66 | <0.001 |
| **Instep** | Constant | -88.53 | 30.13 | -148.20--28.87 | 0.004 | -88.53 | 17.93 | -124.03--53.03 | <0.001 |
|  | Age, years | 0.24 | 0.13 | -0.02-0.50 | 0.066 | 0.24 | 0.09 | 0.06-0.42 | 0.008 |
|  | Height, cm | 0.49 | 0.16 | 0.17-0.80 | 0.003 | 0.49 | 0.10 | 0.29-0.68 | <0.001 |
| **Lateral malleolus** | Constant | -63.35 | 10.20 | -83.54--43.15 | <0.001 | -63.35 | 13.11 | -89.32--37.38 | <0.001 |
|  | Age, years | 0.23 | 0.04 | 0.14-0.32 | <0.001 | 0.23 | 0.04 | 0.14-0.32 | <0.001 |
|  | Height, cm | 0.34 | 0.05 | 0.23-0.45 | <0.001 | 0.34 | 0.08 | 0.18-0.50 | <0.001 |
| **Wrist** | Constant | -7.33 | 9.78 | -26.69-12.04 | 0.455 | -7.33 | 6.58 | -20.35-5.70 | 0.268 |
|  | Age, years | 0.03 | 0.04 | -0.05-0.11 | 0.558 | 0.03 | 0.02 | -0.02-0.07 | 0.295 |
|  | Height, cm | 0.04 | 0.05 | -0.06-0.15 | 0.394 | 0.04 | 0.04 | -0.03-0.11 | 0.217 |

^1^ Standard Koenker and Bassett method; ^2^ Bootstrap resampling (number of replications: 2000); RC: regression coefficient; SE: standard error; CI: confidence interval
