# Supplementary material for: Vibration threshold in non-diabetic subjects
Source: PLoS One. 2020 Oct 7;15(10):e0237733. doi: 10.1371/journal.pone.0237733 (PMC7540842; doi:10.1371/journal.pone.0237733)
Supplement: Attachment — Submitted filename: Response to reviewers.pdf [file pone.0237733.s005.pdf]

<u>Response to the reviewers' comments</u>

Reviewer #1

We thank the reviewer for the comments on our manuscript; we have the impression that the manuscript clearly improved by implementing the suggestions.

<u>Comment 1:</u>

Please shorten the introduction.

<u>Response to comment 1:</u>

As you suggested we shortened the introduction by leaving out information on the qualitative 128-Hz tuning fork as this does not add vital background information for the current study. The current study focusses on determining vibration perception thresholds (VPT) and only a Rydell-Seiffer tuning fork and a biothesiometer are relevant devices that can measure VPT. Consequently, the introduction appears now more coherent.

<u>Comment 2:</u>

Please highlight the practical implications.

<u>Response to comment 2:</u>

We underlined the practical implications in the introduction by adding information on the need for normal values of VPT to follow patients that are treated for their large myelinated nerve fiber deficits (see manuscript lines 68-69). Moreover, we now address the clinical implications in the discussion, by mentioning the possible increase in prevalence of peripheral neuropathies due to population ageing (see manuscript lines 312-316). This underscores the importance of normal values for VPT to enable monitoring of these patients in an adequate and non-invasive way.

Reviewer #2

We thank the reviewer for the constructive comments that helped us to clarify a number of aspects of our manuscript.

<u>Comment 1:</u>

The title of the paper is somewhat misleading since the Authors show that a large proportion of the subjects were not healthy although they did not have diabetes. My suggestion is to remove the word "healthy" from the title.

<u>Response to comment 1:</u>

We agree with your comment and adjusted the title as suggested.

<u>Comment 2:</u>

In the introduction the authors mention that the normal values have been presented previously by (at least) two groups (references 7 and 8). The paper by Bloom & al. was published already 1984 and examined vibration thresholds in 519 patients with varying ages from below 10 to up to 90 years! They do provide mean and range of vibration perception thresholds in different age groups at 3 different locations .Table II Bloom et. al.. The same is true for Maffei et. al. who unfortunately fail to describe which location was used. (I have contacted the authors but have not received any feedback) Consequently, the claim that Bloom et. al. did not specify the normal values (page 3 line 69) is not true, and the current paper is the third one investigating the same questions. The novelty of this paper is that it gives the normal values in two locations not used previously (Instep and Wrist) and that the normal values are presented in microns, rather than Volts, which makes it difficult to compare with previous studies.

<u>Response to comment 2:</u>

We agree that the explanation of the need for revisited normal values was unclear. It is correct that Bloom et al. already provided reference values for VPT. However, as already mentioned shortly by Bloom et al. and confirmed by other studies, height seems to be another important predictor to take into account next to age. Maffei et al. considered height as well as age but did not specify measurement locations. As a consequence, there are no optimal normal values for a clinical setting yet. In the current study, we provide normal values dependent on age and height; these normal values are of major importance in clinical practice, to follow up patients with large myelinated nerve

fiber deficits. In response to your comment, we added a more clear description of the lack of normal values based on age and height and the need for revised normal values to the introduction (see manuscript lines 63-69).

Comment 3:
Introduction page 3 line 60-62: "By using the biothesiometer and thus detecting the first sensation of vibration, adaption to the sensation of vibration is less likely to develop." – This needs a reference.
Response to comment 3:
Thank you for this suggestion. Accordingly, we added a reference for the argument that adaption to the sensation of vibration is less likely to develop when using a biothesiometer (see manuscript lines 56-57).

Comment 4:
Page 3 line 62-63: "recent study has shown that a handheld biothesiometer is a superior tool to monitor changes in vibration sense" – should be: "superior to Rydel–Seiffer tuning fork" (the paper compares only these two methods).
Response to comment 4:
We agree: a handheld biothesiometer is a superior tool compared to a Rydel-Seiffer tuning fork (see manuscript lines 57-60). We changed the sentence accordingly.

Comment 5:
It seems that the authors had full access to medication history. Since 54.3 % of the patients used some medication and the mean age was high it would be interesting to know how many were using statins. This might influence the results (see e.g. Svendsen TK et al. Statin use and peripheral nerve function-A prospective follow-up study. Basic Clin Pharmacol Toxicol. 2019 Sep 10. 10.1111/bcpt.13320)
Response to comment 5:
Thank you for this suggestion. Accordingly, we added information on the use of statins (see manuscript Table 2). We also cited the suggested reference (reference 13 in the reference list) and an additional reference (reference 14 in the reference list). Because anti-retroviral medication is also well known for its adverse effects on peripheral nerve function, we have also added information on the use of this medication (none of the participants used such treatment) and an appropriate reference (reference 15 in the reference list).

Comment 6:
How many patients did you have in each group? Our experience is that the variability increases dramatically after the age of 65.
Response to comment 6:
Thank you for mentioning your observation. We included information on the number of subjects per age and height group in Table 7.
And indeed, the increase in variability can also be observed in the current study as well as an increase in the variability of VPT with increasing height (see manuscript Table 8).

Comment 7:
As the authors mention the limitations of biothesiometer are reaction time of the subject and the ability to concentrate. Our experience measuring vibration perception thresholds with a different kind of device that can visualize the vibration perception thresholds is that approximately 5-10 % of the patients have difficulties in performing the measurement correct and these persons often report vibration thresholds that are too good, sometimes impossible to be true. My question is how many of the subjects had to do more than two measurements (deviation more that 20%) and why did you choose to use the lowest threshold? I would suggest that you try to exclude all the measurement where the subject seems to be guessing and calculate the mean between the two measurements if possible.
Response to comment 7:
Considering the recording of the vibration perception, it remains a limitation of sensory testing that the measurements stay subjective and depend on the focus and reaction time of the subject (see manuscript lines 299-303). Therefore, to prevent other possible influencing factors, the study protocol of the current study was standardized (see manuscript lines 291-296): measurements were

performed in the same room, at similar room temperature and were repeated provided the first two measurements differed more than 20%. In response to your comment, we added information on the number of subjects that underwent a third measurement to Table 1. Furthermore, we changed the method and used the mean of all included measurements as you suggested (see manuscript lines 111-112). In the supporting information, we added a sensitivity analysis in which all subjects that underwent a third measurement were excluded and the mean between the two remaining measurements was calculated (see supporting information S2). Age and height were confirmed as independent determinants of VPT and the regression coefficients remained comparable to the ones calculated without excluding subjects that underwent a third measurement. Furthermore, we added a comparison of clinical characteristics between the group without a third measurement and the group that underwent a third measurement (see supporting information Table S2.2).

Comment 8:

Although the regression equations give the mean values at different locations (or logarithm of the mean VPT?), more interesting and correct would be to present centile charts as Bloom et. Al. According to Bloom (and to our data) the variability of VPTs increases with increasing age and the confidence intervals should be presented for different age groups.

Response to comment 8:

Thank you for this suggestion. Accordingly, we added Table 8 which presents the mean VPT and 95% confidence intervals for the different age and height groups.

The main difference compared to the study by Bloom et al. is that we also included height as a predictor of VPT because both height and age were independent predictors of VPT. Therefore, we added quantile charts of VPT as a function of age (see manuscript Figure 6) and height (see manuscript Figure 7). To include the respective second predictor in the chart, age and height were included as points with a color gradient. These colored points represent the participants included in the current study and are clustered around the quantiles which were calculated for the first predictor (age or height, respectively).

To be able to calculate normal values in clinical practice for every individual patient, we added nomograms in the supporting information which are based on the regression equations. These regression equations include the coefficients of the 95th percentile which was chosen as the cut-off for the normal value. By filling in the age and height of the patient on the outer scales of the nomograms and connecting the two points by drawing a straight line, it will be possible to read the normal value for the VPT for the respective patient from the middle scale.

Comment 9:

It could also benefit if the data would be presented as medians+ confidence intervals for different age groups.

Response to comment 9:

As suggested the data is now presented as mean VPT and 95% confidence interval for different age and height groups in Table 8.
