# Supplementary material for: Vibration threshold in non-diabetic subjects
Source: PLoS One. 2020 Oct 7;15(10):e0237733. doi: 10.1371/journal.pone.0237733 (PMC7540842; doi:10.1371/journal.pone.0237733)
Supplement: Attachment — Submitted filename: PONE-D-20-09300_reviewer.pdf [file pone.0237733.s006.pdf]

# PLOS ONE

## Vibration thresholds in non-diabetic subjects

--Manuscript Draft--

| | |
|---|---|
| **Manuscript Number:** | PONE-D-20-09300 |
| **Article Type:** | Research Article |
| **Full Title:** | Vibration thresholds in non-diabetic subjects |
| **Short Title:** | Vibration thresholds in non-diabetic subjects |
| **Corresponding Author:** | Svea Nolte<br>Universitair Medisch Centrum Groningen<br>Groningen, NETHERLANDS |
| **Keywords:** | vibration threshold; biothesiometer; large myelinated nerve fiber deficits; peripheral neuropathies |
| **Abstract:** | Measuring vibration perception threshold (VPT) accurately classifies and quantifies the severity of loss of vibration perception. A biothesiometer (Bio-thesiometer®; Bio Medical Instrument Co, Ohio, USA) appears to be the most suitable tool to determine VPT due to its low inter-rater variability and low occurence of adaption to the sensation. Different VPT values for a biothesiometer have been described, however, specification on age, height and different measurement locations is currently lacking. The objective of our study is to identify determinants of vibration perception in non-diabetic subjects, in order to provide individualized normal values of VPTs for clinical practice. Measurements of the vibration perception were performed on the big toes, insteps, lateral malleoli, and wrists. A total of 205 healthy subjects were included (108 (52.7%) males) with a median [interquartile range] age of 59 [51;64] (range 21-80) years. Mean height was 174.45 ± 9.20 cm and mean weight was 82.94 ± 14.84 kg, resulting in a mean BMI of 27.19 ± 4.00 kg/m2. In stepwise forward linear regression analyses, age (st. $\beta = 0.51$, $p < 0.001$) and height (st. $\beta = 0.43$, $p < 0.001$) were found to be the independent unmodifiable determinants of the VPT at the big toe. Regression coefficients for quantiles of the determinants age and height were incorporated in the corresponding regression equations. This study provides equations to calculate age- and height-specific normal values for VPT that can be used in clinical practice and in large research studies. |
| **Order of Authors:** | Svea Nolte |
| | Marco van Londen |
| | Jan Willem J. Elting |
| | Bianca T.A. de Greef |
| | Jan B.M. Kuks |
| | Catharina G. Faber |
| | Ilja M. Nolte |
| | Rob J.M. Groen |
| | Stephan J.L. Bakker |
| | Dion Groothof |
| | Ivonne Lesman-Leegte |
| | Stefan P. Berger |
| | Gea Drost |
| **Additional Information:** | |
| **Question** | **Response** |
| **Financial Disclosure** | The author(s) received no specific funding for this work. |

| | |
|---|---|
| Enter a financial disclosure statement that describes the sources of funding for the work included in this submission. Review the submission guidelines for detailed requirements. View published research articles from *PLOS ONE* for specific examples.<br><br>This statement is required for submission and **will appear in the published article** if the submission is accepted. Please make sure it is accurate.<br><br>**Unfunded studies**<br>Enter: *The author(s) received no specific funding for this work.*<br><br>**Funded studies**<br>Enter a statement with the following details:<br>• Initials of the authors who received each award<br>• Grant numbers awarded to each author<br>• The full name of each funder<br>• URL of each funder website<br>• Did the sponsors or funders play any role in the study design, data collection and analysis, decision to publish, or preparation of the manuscript?<br>• **NO** - Include this sentence at the end of your statement: *The funders had no role in study design, data collection and analysis, decision to publish, or preparation of the manuscript.*<br>• **YES** - Specify the role(s) played.<br><br>* typeset | |
| **Competing Interests**<br><br>Use the instructions below to enter a competing interest statement for this submission. On behalf of all authors, disclose any competing interests that could be perceived to bias this work—acknowledging all financial support and any other relevant financial or non-financial competing interests.<br><br>This statement **will appear in the published article** if the submission is accepted. Please make sure it is accurate. View published research articles from *PLOS ONE* for specific examples. | The authors have declared that no competing interests exist. |

Powered by Editorial Manager® and ProduXion Manager® from Aries Systems Corporation

| | |
|---|---|
| **NO authors have competing interests**<br><br>Enter: *The authors have declared that no competing interests exist.*<br><br>**Authors with competing interests**<br><br>Enter competing interest details beginning with this statement:<br><br>*I have read the journal's policy and the authors of this manuscript have the following competing interests: [insert competing interests here]*<br><br>* typeset | |
| **Ethics Statement**<br><br>Enter an ethics statement for this submission. This statement is required if the study involved:<br><br>• Human participants<br>• Human specimens or tissue<br>• Vertebrate animals or cephalopods<br>• Vertebrate embryos or tissues<br>• Field research<br><br>Write "N/A" if the submission does not require an ethics statement.<br><br>General guidance is provided below. Consult the submission guidelines for detailed instructions. **Make sure that all information entered here is included in the Methods section of the manuscript.** | The study was approved by the local Medical Ethical committee (METc 2014/077). All procedures are in accordance with the declaration of Helsinki. |

Powered by Editorial Manager® and ProduXion Manager® from Aries Systems Corporation

| | |
|---|---|
| **Format for specific study types**<br><br>**Human Subject Research (involving human participants and/or tissue)**<br>• Give the name of the institutional review board or ethics committee that approved the study<br>• Include the approval number and/or a statement indicating approval of this research<br>• Indicate the form of consent obtained (written/oral) or the reason that consent was not obtained (e.g. the data were analyzed anonymously)<br><br>**Animal Research (involving vertebrate animals, embryos or tissues)**<br>• Provide the name of the Institutional Animal Care and Use Committee (IACUC) or other relevant ethics board that reviewed the study protocol, and indicate whether they approved this research or granted a formal waiver of ethical approval<br>• Include an approval number if one was obtained<br>• If the study involved *non-human primates*, add *additional details* about animal welfare and steps taken to ameliorate suffering<br>• If anesthesia, euthanasia, or any kind of animal sacrifice is part of the study, include briefly which substances and/or methods were applied<br><br>**Field Research**<br><br>Include the following details if this study involves the collection of plant, animal, or other materials from a natural setting:<br>• Field permit number<br>• Name of the institution or relevant body that granted permission | |
| **Data Availability**<br><br>Authors are required to make all data underlying the findings described fully available, without restriction, and from the time of publication. PLOS allows rare exceptions to address legal and ethical concerns. See the PLOS Data Policy and FAQ for detailed information. | No - some restrictions will apply |

Powered by Editorial Manager® and ProduXion Manager® from Aries Systems Corporation

| | |
|---|---|
| A Data Availability Statement describing where the data can be found is required at submission. Your answers to this question constitute the Data Availability Statement and **will be published in the article**, if accepted.<br><br>**Important:** Stating 'data available on request from the author' is not sufficient. If your data are only available upon request, select 'No' for the first question and explain your exceptional situation in the text box.<br><br>Do the authors confirm that all data underlying the findings described in their manuscript are fully available without restriction? | |
| **Describe where the data may be found in full sentences. If you are copying our sample text, replace any instances of XXX with the appropriate details.**<br><br>• If the data are **held or will be held in a public repository**, include URLs, accession numbers or DOIs. If this information will only be available after acceptance, indicate this by ticking the box below. For example: *All XXX files are available from the XXX database (accession number(s) XXX, XXX.)*.<br>• If the data are all contained **within the manuscript and/or Supporting Information files**, enter the following: *All relevant data are within the manuscript and its Supporting Information files.*<br>• If neither of these applies but you are able to provide **details of access elsewhere**, with or without limitations, please do so. For example:<br><br>*Data cannot be shared publicly because of [XXX]. Data are available from the XXX Institutional Data Access / Ethics Committee (contact via XXX) for researchers who meet the criteria for access to confidential data.*<br><br>*The data underlying the results presented in the study are available from (include the name of the third party* | Data cannot be shared publicly because of the sensitivity of the data and the restrictions from the informed consent. Data are available from the Institutional Data Access / Ethics Committee (contact via corresponding author) for researchers who meet the criteria for access to confidential data. |

| | |
|---|---|
| *and contact information or URL).*<br>• This text is appropriate if the data are owned by a third party and authors do not have permission to share the data.<br><br>* typeset | |
| Additional data availability information: | |

Powered by Editorial Manager® and ProduXion Manager® from Aries Systems Corporation

Manuscript

Click here to access/download;Manuscript;Manuscript VPT PLOS ONE.docx

# Vibration threshold in non-diabetic subjects

Svea Nolte[1*]; Marco van Londen[2]; Jan Willem J. Elting[3]; Bianca T.A. de Greef[4,5]; Jan B.M. Kuks[3]; Catharina G. Faber[4]; Ilja M. Nolte[6]; Rob J.M. Groen[1]; Stephan J.L. Bakker[2]; Dion Groothof[2]; Ivonne Lesman-Leegte[1]; Stefan P. Berger[2]; and Gea Drost[1,3].

[1] Department of Neurosurgery, University Medical Center Groningen and University of Groningen, Groningen, the Netherlands

[2] Department of Internal Medicine, Division of Nephrology, University Medical Center Groningen and University of Groningen, Groningen, the Netherlands

[3] Department of Neurology, University Medical Center Groningen and University of Groningen, Groningen, the Netherlands

[4] Department of Neurology, Maastricht University Medical Center and University of Maastricht, Maastricht, the Netherlands

[5] Klinische Epidemiologie en Medical Technology Assessment (KEMTA), Maastricht University Medical Center, Maastricht, the Netherlands

[6] Department of Epidemiology, University Medical Center Groningen and University of Groningen, Groningen, the Netherlands

*Corresponding author

E-mail: s.nolte@umcg.nl (SN)

# Abstract

Measuring vibration perception threshold (VPT) accurately classifies and quantifies the severity of loss of vibration perception. A biothesiometer (Bio-thesiometer®; Bio Medical Instrument Co, Ohio, USA) appears to be the most suitable tool to determine VPT due to its low inter-rater variability and low occurence of adaption to the sensation. Different VPT values for a biothesiometer have been described, however, specification on age, height and different measurement locations is currently lacking. The objective of our study is to identify determinants of vibration perception in non-diabetic subjects, in order to provide individualized normal values of VPTs for clinical practice. Measurements of the vibration perception were performed on the big toes, insteps, lateral malleoli, and wrists. A total of 205 healthy subjects were included (108 (52.7%) males) with a median [interquartile range] age of 59 [51;64] (range 21-80) years. Mean height was 174.45 ± 9.20 cm and mean weight was 82.94 ± 14.84 kg, resulting in a mean BMI of 27.19 ± 4.00 kg/m$^2$. In stepwise forward linear regression analyses, age (st. $\beta = 0.51$, $p < 0.001$) and height (st. $\beta = 0.43$, $p < 0.001$) were found to be the independent unmodifiable determinants of the VPT at the big toe. Regression coefficients for quantiles of the determinants age and height were incorporated in the corresponding regression equations. This study provides equations to calculate age- and height-specific normal values for VPT that can be used in clinical practice and in large research studies.

# Introduction

Vibration sense is often impaired in large myelinated nerve fiber deficits such as in diabetic polyneuropathy [1,2].

To examine vibration sense in a quantitative way vibration perception thresholds (VPTs) can be determined. VPT is defined as the lowest vibration energy that can be perceived [5]. The VPT can be either determined using a Rydel-Seiffer graduated tuning fork or a biothesiometer, which are both designed to measure deep sensation [2,6]. The VPT determined using the Rydel-Seiffer tuning fork is defined as the value nearest to the point where vibration is no longer perceived [2]. In contrast to the Rydel-Seiffer graduated tuning fork, a biothesiometer measures the VPT as the point at which the first sensation of the vibration appears [6]. By using the biothesiometer and thus detecting the first sensation of vibration, adaption to the sensation of vibration is less likely to develop [5]. Moreover, a recent study has shown that a handheld biothesiometer is a superior tool compared to the Rydel-Seiffer graduated tuning fork to monitor changes in vibration sense over time, which makes it ideal for long-term follow-ups [5]. It is also noteworthy that a biothesiometer has a low inter-rater variability [7], particularly important in clinical practice and in large studies in which several investigators perform the measurements.

Different VPT values for a biothesiometer have been described [7,8] and it appeared that in individuals without sensory disturbances VPT are not only influenced by age [7] but also by height [8]. Bloom et al. already mentioned that next to a strong correlation with age, height appeared to be of influence on VPT assessed at the feet [7]. Maffei et al. provided normal values depending on age and height but did not specify the location of the measurement [8]. As therapeutic approaches for large myelinted nerve fiber deficits are proceeding [9], specific normal values to follow up patients are crucial.

In this study, we aimed to provide individualized normal values for VPT in a group of non-diabetic subjects for different measurement locations on the body which can be used as reference values for clinical use.

# Materials and methods

## Participants

The inclusion of healthy subjects is displayed in Fig 1.

In this study, 260 subjects, during their screening for living kidney donation, were assessed for eligibility. Subjects were at least 18 years of age. We excluded subjects with vitamin B12 deficiency [10], alcohol intake of more than three units per day, missing blood values of vitamin B12, fasting plasma glucose or HbA1c, and patients with diabetes mellitus. Diabetes was defined as fasting plasma glucose higher or equal to 7.0 mmol/L, HbA1c above or equal to 48 mmol/mol, or the use of antidiabetic medication [11]. Other exclusion criteria were neurological disorders, trauma to limbs, and malignancies. However, these were not present in our subject population. All subjects underwent measurements in the University Medical Center Groningen (UMCG) as part of the TransplantLines Biobank and Cohort study, in which the living kidney donors serve as healthy controls [12]. We collected data on age, sex, height, weight, medical history, medication use [13,14,15], and co-morbidities from hospital records of the donor screening. Subjects were included between June 2016 and October 2017. All participants provided written informed consent. The study was approved by the local Medical Ethical committee (METc 2014/077). All procedures are in accordance with the declaration of Helsinki.

**Fig 1. Participants.**

## Recording of vibration perception

To measure the vibration sensation in a group of non-diabetic subjects, we used a handheld biothesiometer (Bio-thesiometer®; Bio Medical Instrument Co, Ohio, USA) with a rubber applicator, which is the part that vibrates at a frequency of 120 Hz and with an amplitude range of 0.01-25.5 µm. The vibration is not audible [6]. The measurements were performed in the morning by a group of 26 well-trained investigators. Before the measurements were performed, subjects were familiarized with the sensation of a biothesiometer by applying the vibration button to a hand, while increasing the amplitude of the vibrator button from zero to the highest amplitude possible. For the measurements, the subjects were lying on a bed after taking off their socks. During the measurements, the vibrator button was primarily applied to the big toes and secondarily to three other points of the body, i.e. the insteps, lateral malleoli, and wrists (Fig 2). Subjects were asked to concentrate on feeling the vibration sensation and to report the first sensation of vibration by saying “Stop”. Each so called ‘threshold measurement’ was tested bi-laterally two times (Fig 3). If the difference between the first two measurements at one side was more than 20%, the point was tested for a third time. If this was the case, the most deviating value was excluded. More detailed information on the number of subjects that underwent a third measurement is shown in Table 1. The mean value of all included measurements is defined as the threshold at the given point of measurement, used to determine the VPT. To translate the voltage readings obtained from the Biothesiometer scale into absolute amplitude values the calibration table of the manual was used [6]. The VPT are given in microns of motion in which a micron equalizes $10^{-4}$ cm [6].

Additionally, in order to evaluate the inter-rater reliability of a biothesiometer, a group of 41 subjects underwent three repeated measures, each performed by a different investigator.

**Table 1. Details on number of third measurement[1].**

| Big toes | | Insteps | | Lateral malleoli | | Wrists | |
|---|---|---|---|---|---|---|---|
| Left | Right | Left | Right | Left | Right | Left | Right |
| 32 | 26 | 47 | 29 | 35 | 34 | 20 | 13 |

[1]A third measurement was performed if the difference between the first two measurements at one side was >20%.

**Fig 2. Measured points (big toe, instep, lateral malleolus, wrist).**

**Fig 3. Measurement.**

## Blood samples

We collected data on fasting blood glucose, HbA1c, folic acid, and vitamin B12 concentrations. Analyses of fasting blood glucose and vitamin B12 were both done using a Roche Cobas 8000 platform (Roche, Mannheim, Germany). HbA1c levels were determined with a Tosoh G8 (ion exchange HPLC) (Tosoh/Sysmex Europe, Norderstedt, Germany).

## Statistical analysis

Descriptive statistics were used to characterize the study population. Continuous variables with a normal distribution were displayed as mean (standard deviation, SD). Continuous variables with a skewed distribution were displayed as median [interquartile range, IQR]. Categorical variables were displayed as frequencies with percentages. Distributions of continuous variables were checked with histograms, QQ plots, and Shapiro-Wilk test.  As no clinical relevant side-to-side variation was found, it was decided to define the mean of all included measurements of both sides as threshold.

To identify clinical characteristics that function as unmodifiable determinants of the VPT, stepwise forward linear regression analyses were performed for the measurements at the big toes. The variables age, height, sex, weight, and smoking behaviour were tested. The probablility-of-F-to-enter was defined as $\leq 0.05$ and the probability-of-F-to-remove as $\geq 0.1$. To conduct linear regression analyses the obtained thresholds were log transformed because of non normal distributions.

By means of quantile regression models using the 95th quantile corresponding regression, equations were established to be able to calculate a normal VPT for each individual person dependent on all significant predictors. The 95th quantile corresponds to the 95% specificity. Regression equations were computed for all points of measurement: big toes, insteps, lateral malleoli, and wrists. Previous studies used quantile regressions to establish a corresponding regression equation [16,17]. To assess the accuracy of the quantile regression model, the standard Koenker and Bassett method as well as bootstrap resampling with performing 2000 replications were conducted to provide standard errors [18]. When calculating the normal VPT values using the derived equations, the result will be given in microns of motion [6]. To determine the applicability of these equations the fifth percentile of age was used as a cut-off because of the few young subjects present in the study population. In the age group younger than 39 years only few subject per age were included and therefore the generalizability of these measurements seems not reliable. Consequently these equations cannot be used for subjects younger than 39 years.

To explore the inter-rater reliability of a biothesiometer an intraclass correlation coefficient (ICC) was performed. ICC estimates and their 95% confident intervals were calculated based on a single rating, absolute agreement, and two-way random effects model ($ICC_{2,1}$). ICCs were interpreted along the following cut-offs: >0.9, excellent reliability; 0.75-0.9, good reliability; 0.5-0.75 moderate reliability; and <0.5, poor reliability [19].

SPSS version 23 for Windows (IBM, Armonk, USA), Stata 15 (StataCorp LCC, College Station, USA) and Graphpad Prism 7 for Windows (Graphpad, San Diego, CA), Python 3.0 (Python Software Foundation, Amsterdam, Netherlands) were used for statistical analyses. P-values below 0.05 were considered statistically significant.

# Results

## Participant characteristics

The mean and median of clinical characteristics of the 205 included subjects are summarized in Table 2. The subject age was 59 [51;64] years and 108 (52.7%) subjects were male. Their height and weight were 174.45 ± 9.20 cm and and 82.94 ± 14.84 kg respectively, resulting in a BMI of 27.19 ± 4.00 kg/m2. Of the subjects, 56 (27.3%) had hypertension, 15 (7.3%) had a history of cardiovascular disease, 112 (54.6%) used medication and 28 (13.7%) were current smokers. Alcohol intake was 0.47 [0;0.86] units per day. Blood glucose was 5.40 ± 0.54 mmol/L, HbA1c was 37 [35;39] mmol/mol (5.5 [5.4;5.7] %), vitamin B12 was 298.0 [234.0;371.5] pmol/L, and folic acid was 15.1 [11.4;22.0] nmol/L.

**Table 2. Clinical characteristics of the cohort.**

| **Variable** | | **Range** |
|---|---|---|
| Number | 205 | |
| Age, years | 59 [51;64] | 21-80 |
| Sex, n(%) male | 108 (52.7) | |
| Height, cm | 174.45 ± 9.20 | 152.50-195.50 |
| Weight, kg | 82.94 ± 14.84 | 54.00-123.80 |
| BMI, kg/m$^2$ | 27.19 ± 4.00 | 18.90-40.90 |
| Systolic blood pressure, mmHg | 127.32 ± 15.05 | 93.00-175.00 |
| Diastolic blood pressure, mmHg | 74.90 ± 10.93 | 49.00-100.00 |
| Hypertension, n(%) | 56 (27.3) | |
| History of cardiovascular disease, n(%) | 15 (7.3) | |
| Medication use, n(%) | 112 (54.6) | |
| Statins, n(%) | 20 (9.8) | |
| Antiretroviral medication, n(%) | 0 (0) | |
| Smoking, n(%) | 28 (13.7) | |
| Alcohol intake, units/day | 0.47 [0;0.86] | 0-2.86 |
| *Relevant blood values* | | |
| Blood glucose, mmol/L | 5.40 ± 0.54 | 3.70-6.80 |
| HbA1c, mmol/mol | 37 [35;39] | 30-47 |
| Vitamin B12, pmol/L | 298.0 [234.0;371.5] | 146.0-750.0 |
| Folic acid, nmol/L | 15.1 [11.4;22.0] | 3.2-45.4 |

## Determinants of the VPT

The results of the analyses of determinants of VPT at the big toe are summarized in Table 3. In stepwise forward linear regression analyses, age (st. $\beta = 0.51$, $p < 0.001$) and height (st. $\beta = 0.43$, $p < 0.001$) were significantly associated with VPT at the big toe, $F(2,202) = 52.724$, $p < 0.001$, $R^2 = 0.343$. As independent determinants, age and height were as unmodifiable factors included in the regression equations to calculate the normal values of the VPT.

The results of the linear regression analyses at the other points of measurement are shown in Table 4, 5, and 6.

**Table 3. Forward selection multiple linear regression models of determinants of VPT at the big toe[1].**

| Independent variable | B (95% CI) | Standardized *β* | P-value |
|---|---|---|---|
| Age, years | 0.03 (0.02-0.03) | 0.51 | <0.001 |
| Height, cm | 0.03 (0.02-0.03) | 0.43 | <0.001 |

[1] Forward selection, probability-of-F-to-enter ≤ 0.05, probability-of-F-to-remove ≥ 0.1
CI: confidence interval

**Table 4. Forward selection multiple linear regression analysis of determinants of VPT at the insteps[1].**

| Independent variable | B (95% CI) | Standardized *β* | P-value |
|---|---|---|---|
| Age, years | 0.03 (0.02-0.03) | 0.50 | <0.001 |
| Height, cm | 0.02 (0.01-0.03) | 0.30 | <0.001 |
| Weight, kg | 0.01 (0-0.01) | 0.17 | 0.02 |

[1] Forward selection, probability-of-F-to-enter ≤0.05, probability-of-F-to-remove ≥0.1
CI: confidence interval

**Table 5. Forward selection multiple linear regression analysis of determinants of VPT at the lateral malleoli[1].**

| Independent variable | B (95% CI) | Standardized *β* | P-value |
|---|---|---|---|
| Age, years | 0.02 (0.02-0.03) | 0.50 | <0.001 |
| Height, cm | 0.02 (0.01-0.02) | 0.25 | 0.001 |
| Weight, kg | 0.01 (0-0.01) | 0.19 | 0.01 |

[1] Forward selection, probability-of-F-to-enter ≤0.05, probability-of-F-to-remove ≥0.1
CI: confidence interval

**Table 6. Forward selection multiple linear regression analysis of determinants of VPT at the wrists[1].**

| Independent variable | B (95% CI) | Standardized $\beta$ | P-value |
|---|---|---|---|
| Age, years | 0.01 (0.01-0.02) | 0.30 | <0.001 |
| Sex | -0.18 (-0.29--0.08) | -0.23 | 0.001 |

[1] Forward selection, probability-of-F-to-enter ≤0.05, probability-of-F-to-remove ≥0.1
CI: confidence interval

# VPT

Figs 4 and 5 show the relationship between the VPT and the independent unmodifiable determinants of VPT at the big toes, i.e. age and height. The distribution of subjects dependent on age and height and the measured VPT is displayed in Table 7 and 8.

Table 9 provides the unstandardized regression coefficients of the slope for the variables age and height obtained in the multivariable quantile regression model. This model estimates the normal value of the VPT as a function of age and height, with which we can write the corresponding regression equations:

$$\Upsilon_{\mathrm{VPT\_BIGTOE}} = -90.09 + 0.24 \times \mathrm{AGE} + 0.50 \times \mathrm{HEIGHT}$$

$$\Upsilon_{\mathrm{VPT\_INSTEP}} = -98.70 + 0.35 \times \mathrm{AGE} + 0.52 \times \mathrm{HEIGHT}$$

$$\Upsilon_{\mathrm{VPT\_LATMALLEOLUS}} = -70.27 + 0.26 \times \mathrm{AGE} + 0.37 \times \mathrm{HEIGHT}$$

$$\Upsilon_{\mathrm{VPT\_WRIST}} = -4.55 + 0.06 \times \mathrm{AGE} + 0.02 \times \mathrm{HEIGHT}$$

The results of the bootstrapped model are summarized in Table 9. Figs 6 and 7 show conditional quantiles of VPT as a function for age and height for the different measurement locations.

In clinical practice the provided nomograms can be used: by filling in age and height of the patient on the outer scales, connecting the two points by drawing a straight line, the normal value for the VPT can be read from the middle scale (S1 Fig).

**Fig 4. VPT against age for big toes, insteps, lateral malleoli, and wrists.**

**Fig 5. VPT against height for big toes, insteps, lateral malleoli, and wrists.**

**Fig 6. Conditional quantiles of VPT as a function of age for different measurement locations.** Conditional quantiles were modelled flexibly using natural cubic splines of age with two degrees of freedom. Natural boundary conditions were imposed on the 2.5% and 97.5% quantiles of age. The specific quantiles associated with either of the lines is indicated on the right-hand edge of the line. The height across the participants is depicted as a color gradient, with lower values of height given in blue and higher values in red.

**Fig 7. Conditional quantiles of VPT as a function of height for different measurement locations.** Conditional quantiles were modelled flexibly using natural cubic splines of height with two degrees of freedom. The specific quantiles associated with either of the lines is indicated on the right-hand edge of the line. The age across the participants is depicted as a color gradient, with lower values of age given in blue and higher values in red.

**Table 7. Distribution of subjects dependent on age and height.**

| | **Age, years** | | | | | |
|---|---|---|---|---|---|---|
| | ≤ 39 | 40-49 | 50-59 | 60-69 | 70-80 | Total |
| **Number of subjects** | 10 | 39 | 59 | 69 | 28 | 205 |

| | **Height, cm** | | | | | |
|---|---|---|---|---|---|---|
| | <160 | 160-169 | 170-179 | 180-190 | >190 | Total |
| **Number of subjects** | 10 | 56 | 74 | 55 | 10 | 205 |

**Table 8. Measured VPT dependent on age and height grouping.**

| **Age, years** | **Big toes[1]** | **Insteps[1]** | **Lateral malleoli[1]** | **Wrists[1]** |
|---|---|---|---|---|
| ≤ 39 | 0.79 (0.10-1.48) | 0.94 (0.19-1.69) | 0.83 (0.19-1.44) | 0.30 (0.15-0.46) |
| 40-49 | 1.70 (0.73-2.67) | 1.51 (0.81-2.22) | 1.52 (0.91-2.13) | 0.41 (0.34-0.49) |
| 50-59 | 1.92 (1.28-2.58) | 2.66 (1.68-3.65) | 2.36 (1.62-3.10) | 0.48 (0.39-0.57) |
| 60-69 | 2.94 (2.22-3.66) | 3.94 (2.89-5.00) | 3.50 (2.54-4.47) | 0.75 (0.55-0.95) |

| | | | | |
|---|---|---|---|---|
| 70-80 | 3.67 (2.24-5.10) | 4.45 (2.75-6.15) | 3.73 (2.43-5.04) | 0.80 (0.52-1.07) |

[1] mean VPT (95% CI)

| **Height, cm** | **Big toes[1]** | **Insteps[1]** | **Lateral malleoli[1]** | **Wrists[1]** |
|---|---|---|---|---|
| <160 | 1.64 (-0.45-3.73) | 2.11 (-0.53-4.74) | 1.41 (0.68-2.13) | 0.49 (0.33-0.66) |
| 160-169 | 1.57 (1.14-1.99) | 2.07 (1.27-2.87) | 2.17 (1.27-3.07) | 0.52 (0.37-0.67) |
| 170-179 | 1.85 (1.39-2.32) | 2.48 (1.82-3.15) | 2.22 (1.70-2.75) | 0.61 (0.47-0.75) |
| 180-190 | 3.51 (2.53-4.50) | 4.67 (3.33-6.01) | 3.72 (2.67-4.77) | 0.67 (0.47-0.87) |
| >190 | 5.74 (1.40-10.08) | 4.31 (0.33-8.28) | 4.68 (1.23-8.13) | 0.52 (0.28-0.76) |

[1] mean VPT (95% CI)

**Table 9. Quantile regression.**

| | Variable | Original model [1] | | | | Model after bootstrap resampling [2] | | | |
|---|---|---|---|---|---|---|---|---|---|
| | | **RC** | **SE** | **95% CI** | **P-value** | **RC** | **SE** | **95% CI** | **P-value** |
| **Big toe** | Constant | -90.09 | 34.35 | -157.82--22.36 | 0.009 | -90.09 | 15.78 | -121.21--58.98 | 0.006 |
| | Age, years | 0.24 | 0.16 | -0.07-0.54 | 0.132 | 0.24 | 0.09 | 0.07-0.40 | <0.001 |
| | Height, cm | 0.50 | 0.18 | 0.15-0.85 | 0.006 | 0.50 | 0.11 | 0.28-0.71 | <0.001 |
| **Instep** | Constant | -98.70 | 35.76 | -169.21--28.19 | 0.006 | -98.70 | 23.23 | -144.51--52.89 | <0.001 |
| | Age, years | 0.35 | 0.16 | 0.03-0.67 | 0.030 | 0.35 | 0.09 | 0.17-0.54 | <0.001 |
| | Height, cm | 0.52 | 0.19 | 0.16-0.89 | 0.005 | 0.52 | 0.14 | 0.24-0.81 | <0.001 |
| **Lateral malleolus** | Constant | -70.27 | 34.94 | -139.17--1.37 | 0.046 | -70.27 | 13.06 | -96.03--44.52 | <0.001 |
| | Age, years | 0.26 | 0.16 | -0.05-0.57 | 0.100 | 0.26 | 0.07 | 0.13-0.40 | <0.001 |
| | Height, cm | 0.37 | 0.18 | 0.02-0.73 | 0.041 | 0.37 | 0.08 | 0.21-0.54 | <0.001 |
| **Wrist** | Constant | -4.55 | 9.45 | -23.19-14.09 | 0.631 | -4.55 | 5.36 | -15.11-6.02 | 0.397 |
| | Age, years | 0.06 | 0.04 | -0.03-0.14 | 0.164 | 0.06 | 0.02 | 0.02-0.10 | 0.004 |
| | Height, cm | 0.02 | 0.05 | -0.08-0.12 | 0.703 | 0.02 | 0.03 | -0.04-0.08 | 0.525 |

[1] Standard Koenker and Bassett method; [2] Bootstrap resampling (number of replications: 2000); RC: regression coefficient; SE: standard error; CI: confidence interval

## Inter-rater reliability

ICC revealed a good reliability of a biothesiometer measurement at the big toe (ICC = 0.88, 95% CI = 0.81-0.93), the instep (ICC = 0.86, 95% CI = 0.78-0.92), the lateral malleoli (ICC = 0.76, 95% CI = 0.64-0.86), and the wrist (ICC = 0.80, 95% CI = 0.69-0.88).

# Discussion

In this study, we were able to provide individualized normal values for VPT using a biothesiometer which was shown to have a good inter-rater reliability. Moreover, this study showed that VPTs are influenced by age and height which were included when determining the normal values.

We found that with increasing age the vibration perception decreases at all points of measurement. Age-dependency of vibration perception has been first reported by Pearson et al. [20] and has been confirmed by several other studies [2,6-9,21-24]. However, it is not clear what causes this reduction of vibration perception [2]. Vibration is detected by tactile receptors that are located in the epidermis and dermis [25]. When focusing on the detection of vibration, two different receptor systems are known: Pacinian corpuscles and Meissner's corpuscles [25-27]. Both deteriorate and are less present in ageing skin [25,27]. Furthermore, it is known that with ageing myelin alterations take place in peripheral nerves: segmental de- and remyelination, axonal degeneration and regeneration are seen more often. This is possibly due to atherosclerotic vascular changes that happen during aging [28]. Besides atherosclerotic vascular changes, also a generalized reduction in vasculature of the dermis is observed in the elderly [26]. There are also remarkable changes in the central nervous system such as cerebral small vessel disease which are linked to mobility impairment in the elderly [29]. These vascular changes in the central and peripheral nervous system could also influence the

conduction of vibration stimuli. Both, Pacinian corpuscles and Meissner's corpuscles, are supplied by Aβ nerve fibers [27,30]. Demyelination or a decrease in thickness of the myelin possibly causes abnormal Aß-fiber conduction velocities [30]. The above described changes are a possible explanation for the age-dependency of vibration perception.

Height was also an independent determinant: with increasing height the vibration perception decreases at all points of measurement. This height relation can also be observed at the different points of measurement. VPTs at the wrist are lower than at the big toe, instep, or lateral malleolus, which might be linked to a shorter distance between wrist and spinal cord. So far, only Deshpande et al. found height to be an independent determinant of vibration perception [24]. Noteworthy is that according to a recent study, Dutch men are tallest of the world and Dutch women are second tallest after Latvian women [31]. As our study was carried out in the Netherlands, the variation of height within the subject population is likely to be larger than in previously performed studies in other countries. This may add to the association with height that we found.

Strengths of this study include the detailed database in which blood values, medication history and comorbidities are recorded for all subjects. Moreover, the measurements were performed under standardized conditions meaning always in the same environment and location. This is crucial because by applying standardized conditions and providing good training of the different investigators, it is likely that factors that could have influenced the vibration energy, as difference in weight through applying pressure when measuring with a biothesiometer, were ruled out. Moreover, subjects suffering from peripheral neuropathy caused by vitamin B12 deficiency, alcohol abuse or diabetes mellitus were excluded. Furthermore, it was shown that the inter-rater variability of a biothesiometer is low, making it an ideal device for daily clinical practice and large research studies [7]. Nevertheless, it should be noticed that quantitative sensory testing including testing of vibratory stimuli as an objective physical event requires a response of the subject which is a subjective report. As a

consequence, there are limitations inherent to the method due to reaction time of the subject and the ability to focus on the task [32]. One of the weaknesses of our study is the low sample size for the group younger than 39 years. Consequently, it was not possible to establish regression equations that are applicable for this group. When determining vibration perception in clinical practice in a patient younger than 39 years, it is possible to use the normal values for a patient of 39 years. If this patient scores abnormal according to the used normal value, an abnormality in vibration perception is certain. However, a determined normal vibration perception would not be evident. Another weakness of our study is that skin temperature at the different sites of measurement, which possibly affects the vibration perception, was not taken into account [21,22].

Clinical applications of VPT are diseases involving the sensory nerves and especially those primarily affecting large myelinated fibers. Polyneuropathy is one of the most common afflictions. Considering the broad prevalence of polyneuropathy in the population, the continued trend in population ageing, and the increase of incidence with age, age specific thresholds are specifically important [33]. Moreover, it has been reported that presence of polyneuropathy is associated with a significant reduction in quality of life [34]. As polyneuropathy has such a great impact on the life of patients, it is important to examine the affliction in clinical practice by capturing symptoms. Loss of vibration perception can be detected using the regression equations for a biothesiometer that were established in the current subject population. When testing a patient's vibration perception and there is no perception at the big toes, the different equations enable the testing of more proximal locations. The biothesiometer can be applied in daily clinical practice for the quantification of the loss of vibration perception and in large research studies. Larger studies in a younger subject population are needed to extend the results found in this study thereby improving the reliability of the different VPTs. Furthermore, future prospective studies need to validate these regression equations in different healthy subject groups to establish a precise equation

for clinical use. The next step is to test these equations in different patient populations, such as one with diseases like diabetes mellitus, one using neurotoxic medication, or one with abnormal nerve conduction studies.

In conclusion, this study provides normal values for VPT based on unmodifiable determinants, age and height. The established equations can be used to determine loss of vibration perception in clinical practice.

# Acknowledgments

This study was performed using the infrastructure and data provided by the TransplantLines Biobank and Cohort Study, which is registered at ClinicalTrials.gov under Identifier NCT03272841. We thank dr. R. Bakels, neurophysiologist at the UMCG, for advice regarding the content of the manuscript. Moreover, we thank Johan Schneiders for creating the nomograms and Piet H. Toonder, medical photographer at the UMCG, for the visualization of a biothesiometer and the measurements.

# References

1. Tenckhoff HA, Boen FST, Jebsen RH, Spiegler JH. Polyneuropathy in Chronic Renal Insufficiency. JAMA. 1965;192:1121-4.
2. Martina IS, van Koningsveld R, Schmitz PI, van Der Meché FG, van Doorn PA. Measuring Vibration Thresholds with a Graduated Tuning Fork in Normal Aging and in Patients with Polyneuropathy. European Inflammatory Neuropathy Cause and Treatment (INCAT) group. J Neurol Neurosurg Psychiatry. 1998;65(5):743–7.

3. Pourhamidi K, Dahlin LB, Englund E, Rolandsson O. Evaluation of Clinical Tools and Their Diagnostic Use in Distal Symmetric Polyneuropathy. Prim Care Diabetes. 2014;8(1):77–84.
4. Temlett JA. An Assessment of Vibration Threshold Using a Biothesiometer Compared to a C128-Hz Tuning Fork. J Clin Neurosci. 2009;16(11):1435–8.
5. Marcuzzi A, Wainwright AC, Costa DSJ, Wrigley PJ. Vibration Testing: Optimising Methods to Improve Reliability. Muscle Nerve. 2019;59(2):229–35.
6. Bio-Medical Instrument Company. Bio-Thesiometer: Instructions for Operation.
7. Bloom S, Till S, Sönksen P, Smith S. Use Of A Biothesiometer To Measure Individual Vibration Thresholds And Their Variation In 519 Non-Diabetic Subjects. Br Med J (Clin Res Ed). 1984;288(6433):1793–5.
8. Maffei L, Premrou V, Roldan P, Copetti M, Pellegrini F, Rossi MC, Vespasiani G. Vibration Perception Threshold in the Screening of Sensorimotor Distal Symmetric Polyneuropathy: The Need of More Accurate Age-Specific Reference Values. J Diabetes Sci Technol. 2014;8(3):621–2.
9. Callaghan BC, Price RS, Feldman EL. Diagnostic and Therapeutic Advances: Distal Symmetric Polyneuropathy. JAMA. 2015;314(20):2172-81.
10. Roos D. The Vibration Perception Threshold in Gastrectomized Patients with Low Serum B12. A Clinical and Biothesiometric Follow-up after Intensive B12 Therapy. Acta Neurol Scand. 1977;56(6):551–62.
11. Kerner W, Brückel J. Classification And Diagnosis of Diabetes Mellitus. Exp Clin Endocrinol Diabetes. 2014;122(7):384–6.
12. Eisenga MF, Gomes-Neto AW, van Londen M, Ziengs AL, Douwes RM, Stam SP, et al. Rationale and design of TransplantLines: a prospective cohort study and biobank of solid organ transplant recipients. BMJ Open. 2018;8(12):e024502.

13. Svendsen TK, Kroigard T, Wirenfeldt M, Schroder HD, Bak S, Möller S, et al. Statin Use and Peripheral Nerve function – A Prospective Follow-Up Study. Basic Clin Pharmacol Toxicol. 2019:PMID 31505101.
14. Warendorf JK, Vrancken AFJE, van Eijk RPA, Visser NA, van den Berg LH, Notermans NC. Statins Do Not Increase Risk of Polyneuropathy: A Case-Control Study and Literature Review. Neurology. 2019;92(18):e2136-e2144.
15. Stavros K, Simpson DM. Understanding the Etiology and Management of HIV-associated Peripheral Neuropathy. Curr HIV/AIDS Rep. 2014;11(3):195-201.
16. Shirk SD, Mitchell MB, Shaughnessy LW, Sherman JC, Locascio JJ, Weintraub S, Atri A. A Web-Based Normative Calculator for the Uniform Data Set (UDS) Neuropsychological Test Battery. Alzheimers Res Ther. 2011;3(6):32.
17. Sherwood B, Zhou AX, Weintraub S, Wang L. Using Quantile Regression to Create Baseline Norms for Neuropsychological Tests. Alzheimers Dement (Amst). 2015;2:12–8.
18. Rogers WH. Quantile Regression Standard Errors. Stata Tech Bull. 1992;9:16–19.
19. Koo TK, Li MY. A Guideline of Selecting and Reporting Intraclass Correlation Coefficients for Reliability Research. J Chiropr Med. 2016;15(2):155–63.
20. Pearson GHJ. Effect of Age on Vibratory Sensibility. Arch Neurol Psychiatry. 1928;20:482–96.
21. Meh D, Denislic M. Influence of Age, Temperature, Sex, Height and Diazepam on Vibration Perception. J Neurol Sci. 1995;134(1–2):136–42.
22. Tjon-A-Tsien AM, Van Dijk JG, Van Der Velde EA, Kamzoul BA, Lemkes HH. Determinants of Vibration Perception Thresholds in IDDM and NIDDM Patients. Diabetes Res Clin Pract. 1995;27(3):211–9.
23. Lin YH, Hsieh SC, Chao CC, Chang YC, Hsieh ST. Influence of Aging on Thermal and Vibratory Thresholds of Quantitative Sensory Testing. J Peripher Nerv Syst. 2005;10(3):269–81.

24. Deshpande N, Metter EJ, Ling S, Conwit R, Ferrucci L. Physiological Correlates of Age-Related Decline in Vibrotactile Sensitivity. Neurobiol Aging. 2008;29(5):765–73.
25. Verrillo RT. Age Related Changes in the Sensitivity to Vibration. J Gerontol. 1980;35(2):185–93.
26. Cerimele D, Celleno L, Serri F. Physiological Changes in Ageing Skin. Br J Dermatol. 1990;122 Suppl 35:13–20.
27. Paré M, Albrecht PJ, Noto CJ, Bodkin NL, Pittenger GL, Schreyer DJ, et al. Differential Hypertrophy and Atrophy among All Types of Cutaneous Innervation in the Glabrous Skin of the Monkey Hand during Aging and Naturally Occurring Type 2 Diabetes. J Comp Neurol. 2007;501(4):543–67.
28. Knox CA. Neuroanatomical Changes Associated with Aging in the Peripheral Nervous System. In: Albert ML, Knoefel JE, editors. Clinical neurology of aging. 2nd ed. New York: Oxford University Press; 1994. pp. 68–78.
29. Sorond FA, Cruz-Almeida Y, Clark DJ, Viswanathan A, Scherzer CR, De Jager P, et al. Aging, the Central Nervous System, and Mobility in Older Adults: Neural Mechanisms of Mobility Impairment. J Gerontol A Biol Sci Med Sci. 2015;70(12):1526–32.
30. Shaffer SW, Harrison AL. Aging of the Somatosensory System: A Translational Perspective. Phys Ther. 2007;87(2):193–207.
31. NCD Risc Factor Collaboration (NCD-RisC). A Century of Trends in Adult Human Height. Elife. 2016;5:e13410.
32. Shy ME, Frohman EM, So YT, Arezzo JC, Cornblath DR, Giuliani MJ, et al. Quantitative Sensory Testing: Report of the Therapeutics and Technology Assessment Subcommittee of the American Academy of Neurology. Neurology. 2003;60(6):898–904.
33. Visser NA, Notermans NC, Linssen RS, Van Den Berg LH, Vrancken AF. Incidence of Polyneuropathy in Utrecht, the Netherlands. Neurology. 2015;84(3):259–64.

34. Teunissen LL, Eurelings M, Notermans NC, Hop JW, Van Gijn J. Quality of Life in Patients with Axonal Polyneuropathy. J Neurol. 2000;247(3):195–9.

# Supporting Information

**S1 Fig. Nomograms.** By filling in age and height of the patient on the outer scales, connecting the two points by drawing a straight line, the normal value for the VPT (vibration perception threshold) can be read from the middle scale.

**S2. Sensitivity analysis.** Patients who underwent a third measurement were excluded from the study population and the mean between the two included measurements of VPT was calculated. The clinical characteristics are presented in Table S2.1. Table S2.2 provides a comparison of clinical characteristics of the group without a third measurement and the group with a third measurement. Age and height were identified as independent determinants of VPT at the big toes (Table S2.3). The results at the other points of measurement are displayed in Tables S2.4, S2.5, and S2.6. The distribution of subjects dependent on age and height and the measured VPT is displayed in Tables S2.7 and S2.8. The corresponding regression coefficients according to the 95th percentile were calculated and are shown in Table S2.9. The model can be summarized with the following corresponding regression equations:

$$\Upsilon_{VPT\_BIGTOE} = -79.76 + 0.18 \times AGE + 0.45 \times HEIGHT$$

$$\Upsilon_{VPT\_INSTEP} = -88.53 + 0.24 \times AGE + 0.49 \times HEIGHT$$

$$\Upsilon_{VPT\_LATMALLEOLUS} = -63.35 + 0.23 \times AGE + 0.34 \times HEIGHT$$

$$\Upsilon_{VPT\_WRIST} = -7.33 + 0.03 \times AGE + 0.04 \times HEIGHT$$

**Table S2.1. Clinical characteristics of the cohort.**

| **Variable** | | **Range** |
|---|---|---|

| | | |
|---|---|---|
| Number | 120 | |
| Age, years | 57.5 [48;64] | 21-77 |
| Sex, n(%) male | 58 (48.3) | |
| Height, cm | 173.81 ± 9.06 | 152.50-195.50 |
| Weight, kg | 80.78 ± 14.66 | 54.00-118.80 |
| BMI, kg/m$^2$ | 26.63 ± 3.73 | 18.90-35.00 |
| Systolic blood pressure, mmHg | 125.73 ± 16.09 | 93.00-175.00 |
| Diastolic blood pressure, mmHg | 74.35 ± 11.57 | 50.00-100.00 |
| Hypertension, n(%) | 26 (21.7) | |
| History of cardiovascular disease, n(%) | 9 (7.7) | |
| Medication use, n(%) | 61 (51.3) | |
| Statins, n(%) | 13 (10.8) | |
| Antiretroviral medication, n(%) | 0 (0) | |
| Smoking, n(%) | 16 (13.3) | |
| Alcohol intake, units/day | 0 [0;0.70] | 0-2.58 |
| *Relevant blood values* | | |
| Blood glucose, mmol/L | 5.39 ± 0.55 | 3.70-6.80 |
| HbA1c, mmol/mol | 37.00 [34.25;39.00] | 30.00-47.00 |
| Vitamin B12, pmol/L | 308.0 [232.0;373.5] | 146.0-750.0 |
| Folic acid, nmol/L | 15.1 [11.0;21.3] | 3.2-45.4 |

**Table S2.2. Comparison**

| Variable | No $3^{rd}$ measurement | $3^{rd}$ measurement | P-value |
|---|---|---|---|
| Number | 120 | 85 | |
| Age, years | 57.5 [48;64] | 61.0 [55;66] | 0.01 |
| Sex, n(%) male | 58 (48.3) | 50 (58.8) | 0.14 |
| Height, cm | 173.81 ± 9.06 | 175.35 ± 9.36 | 0.24 |
| Weight, kg | 80.78 ± 14.66 | 85.99 ± 14.64 | 0.01 |
| BMI, kg/m$^2$ | 26.63 ± 3.73 | 27.97 ± 4.26 | 0.02 |
| Systolic blood pressure, mmHg | 125.73 ± 16.09 | 129.65 ± 13.14 | 0.07 |
| Diastolic blood pressure, mmHg | 74.35 ± 11.57 | 75.70 ± 9.94 | 0.38 |
| Hypertension, n(%) | 26 (21.7) | 30 (36.1) | 0.03 |
| History of cardiovascular disease, n(%) | 9 (7.7) | 6 (7.2) | 0.90 |
| Medication use, n(%) | 61 (51.3) | 51 (60.7) | 0.18 |
| Statins, n(%) | 13 (10.8) | 7 (8.2) | 0.54 |
| Antiretroviral medication, n(%) | 0 (0) | 0 (0) | 1.00 |
| Smoking, n(%) | 16 (13.3) | 12 (14.1) | 0.87 |
| Alcohol intake, units/day | 0 [0;0.70] | 0.13 [0;0.88] | 0.10 |
| *Relevant blood values* | | | |
| Blood glucose, mmol/L | 5.39 ± 0.55 | 5.42 ± 0.53 | 0.73 |
| HbA1c, mmol/mol | 37.00 [34.25;39.00] | 37.00 [35.00;39.00] | 0.37 |
| Vitamin B12, pmol/L | 308.0 [232.0;373.5] | 294.0 [234.0;359.5] | 0.69 |
| Folic acid, nmol/L | 15.1 [11.0;21.3] | 15.3 [13.3;22.9] | 0.24 |

**Table S2.3. Forward selection multiple linear regression models of determinants of VPT at the big toe[1].**

| Independent variable | B (95% CI) | Standardized $\beta$ | P-value |
|---|---|---|---|
| Age, years | 0.03 (0.02-0.03) | 0.55 | <0.001 |
| Height, cm | 0.03 (0.02-0.04) | 0.51 | <0.001 |

[1] Forward selection, probability-of-F-to-enter ≤ 0.05, probability-of-F-to-remove ≥ 0.1
CI: confidence interval

**Table S2.4. Forward selection multiple linear regression analysis of determinants of VPT at the insteps[1].**

| Independent variable | B (95% CI) | Standardized $\beta$ | P-value |
|---|---|---|---|
| Age, years | 0.02 (0.02-0.03) | 0.49 | <0.001 |
| Height, cm | 0.02 (0.01-0.03) | 0.34 | 0.001 |
| Weight, kg | 0.01 (0-0.01) | 0.19 | 0.05 |

[1] Forward selection, probability-of-F-to-enter ≤0.05, probability-of-F-to-remove ≥0.1
CI: confidence interval

**Table S2.5. Forward selection multiple linear regression analysis of determinants of VPT at the lateral malleoli[1].**

| Independent variable | B (95% CI) | Standardized $\beta$ | P-value |
|---|---|---|---|
| Age, years | 0.02 (0.02-0.03) | 0.51 | <0.001 |
| Height, cm | 0.01 (0-0.03) | 0.28 | 0.01 |
| Sex | -0.21 (-0.39--0.03) | -0.23 | 0.03 |

[1] Forward selection, probability-of-F-to-enter ≤0.05, probability-of-F-to-remove ≥0.1
CI: confidence interval

**Table S2.6. Forward selection multiple linear regression analysis of determinants of VPT at the wrists[1].**

| Independent variable | B (95% CI) | Standardized $\beta$ | P-value |
|---|---|---|---|
| Age, years | 0.01 (0-0.01) | 0.24 | 0.01 |
| Sex | -0.23 (-0.35--0.11) | -0.33 | <0.001 |

[1] Forward selection, probability-of-F-to-enter ≤0.05, probability-of-F-to-remove ≥0.1
CI: confidence interval

**Table S2.7. Distribution of subjects dependent on age and height.**

| | **Age, years** | | | | | |
|---|---|---|---|---|---|---|
| | ≤ 39 | 40-49 | 50-59 | 60-69 | 70-80 | Total |
| **Number of subjects** | 8 | 30 | 32 | 38 | 12 | 120 |

| | **Height, cm** |
|---|---|

| | <160 | 160-169 | 170-179 | 180-190 | >190 | Total |
|---|---|---|---|---|---|---|
| **Number of subjects** | 5 | 35 | 49 | 24 | 7 | 120 |

**Table S2.8. Measured VPT dependent on age and height grouping.**

| **Age, years** | **Big toes[1]** | **Insteps[1]** | **Lateral malleoli[1]** | **Wrists[1]** |
|---|---|---|---|---|
| ≤ 39 | 1.06 (-0.004-2.13) | 1.32 (0.05-2.60) | 1.12 (0.24-2.00) | 0.44 (0.20-0.68) |
| 40-49 | 2.52 (0.84-4.19) | 2.31 (0.95-3.67) | 2.27 (1.20-3.34) | 0.59 (0.46-0.72) |
| 50-59 | 2.61 (1.78-3.46) | 3.59 (1.90-5.27) | 3.04 (1.89-4.19) | 0.72 (0.52-0.91) |
| 60-69 | 2.15 (1.29-3.01) | 2.57 (1.64-3.49) | 2.44 (1.79-3.09) | 0.62 (0.39-0.84) |
| 70-80 | 5.22 (2.84-7.60) | 6.13 (3.36-8.89) | 4.73 (2.83-6.62) | 0.84 (0.49-1.18) |

[1] mean VPT (95% CI)

| **Height, cm** | **Big toes[1]** | **Insteps[1]** | **Lateral malleoli[1]** | **Wrists[1]** |
|---|---|---|---|---|
| <160 | 0.80 (0.45-1.16) | 1.09 (0.77-1.41) | 1.19 (0.70-1.68) | 0.51 (0.31-0.72) |
| 160-169 | 1.95 (1.28-2.61) | 2.15 (1.30-3.00) | 2.07 (1.37-2.80) | 0.57 (0.43-0.71) |
| 170-179 | 2.61 (1.81-3.41) | 3.31 (2.22-4.39) | 3.02 (2.24-3.79) | 0.83 (0.60-1.06) |
| 180-190 | 3.97 (2.23-5.71) | 4.48 (2.53-6.42) | 3.89 (2.74-5.05) | 0.76 (0.56-0.95) |
| >190 | 8.71 (1.41-16.00) | 7.25 (0.12-14.39) | 6.11 (0.33-11.89) | 0.71 (0.30-1.13) |

[1] mean VPT (95% CI)

**Table S2.9. Quantile regression.**

| | Variable | Original model [1] | | | | Model after bootstrap resampling [2] | | | |
|---|---|---|---|---|---|---|---|---|---|
| | | RC | SE | 95% CI | P-value | RC | SE | 95% CI | P-value |
| **Big toe** | Constant | -79.76 | 30.51 | -140.17--19.34 | 0.010 | -79.76 | 16.31 | -112.06--47.45 | <0.001 |
| | Age, years | 0.18 | 0.13 | -0.08-0.44 | 0.177 | 0.18 | 0.08 | 0.03-0.34 | 0.023 |
| | Height, cm | 0.45 | 0.16 | 0.13-0.77 | 0.006 | 0.45 | 0.11 | 0.23-0.66 | <0.001 |
| **Instep** | Constant | -88.53 | 30.13 | -148.20--28.87 | 0.004 | -88.53 | 17.93 | -124.03--53.03 | <0.001 |
| | Age, years | 0.24 | 0.13 | -0.02-0.50 | 0.066 | 0.24 | 0.09 | 0.06-0.42 | 0.008 |
| | Height, cm | 0.49 | 0.16 | 0.17-0.80 | 0.003 | 0.49 | 0.10 | 0.29-0.68 | <0.001 |
| **Lateral malleolus** | Constant | -63.35 | 10.20 | -83.54--43.15 | <0.001 | -63.35 | 13.11 | -89.32--37.38 | <0.001 |
| | Age, years | 0.23 | 0.04 | 0.14-0.32 | <0.001 | 0.23 | 0.04 | 0.14-0.32 | <0.001 |
| | Height, cm | 0.34 | 0.05 | 0.23-0.45 | <0.001 | 0.34 | 0.08 | 0.18-0.50 | <0.001 |
| **Wrist** | Constant | -7.33 | 9.78 | -26.69-12.04 | 0.455 | -7.33 | 6.58 | -20.35-5.70 | 0.268 |
| | Age, years | 0.03 | 0.04 | -0.05-0.11 | 0.558 | 0.03 | 0.02 | -0.02-0.07 | 0.295 |
| | Height, cm | 0.04 | 0.05 | -0.06-0.15 | 0.394 | 0.04 | 0.04 | -0.03-0.11 | 0.217 |

[1] Standard Koenker and Bassett method; [2] Bootstrap resampling (number of replications: 2000); RC: regression coefficient; SE: standard error; CI: confidence interval

# Financial Disclosure Statement

This research received no external funding.

# Competing Interests

None of the authors has any conflict of interest to disclose.

# Data availability

The datasets generated during and/or analyzed during the current study are not publicly available due to the sensitivity of the data and the restrictions from the informed consent but are available from the corresponding author on reasonable request.

Figure 1

Click here to access/download;Figure;Fig 1.tif

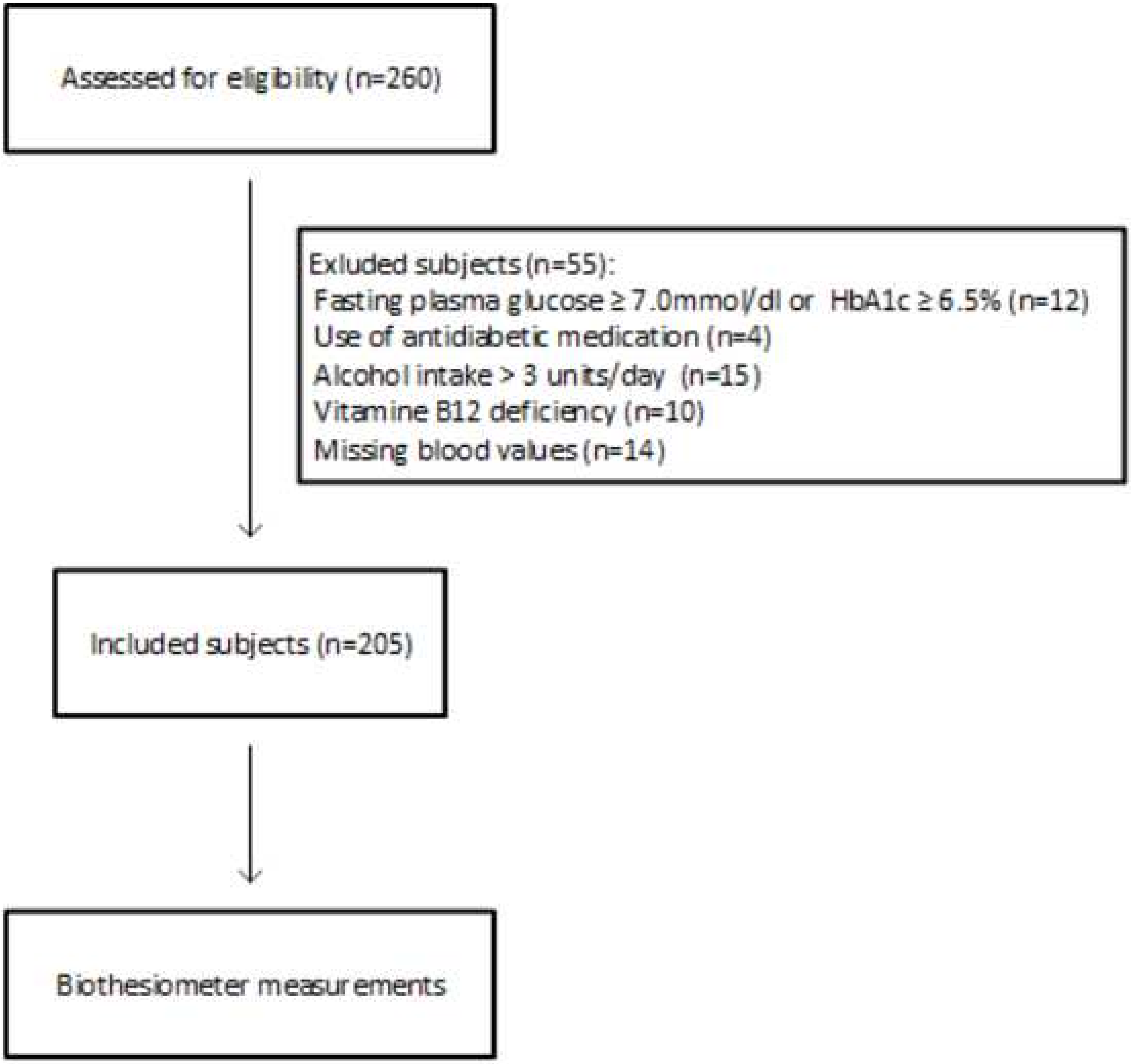

Figure 2

Click here to access/download;Figure;Fig 2.tif

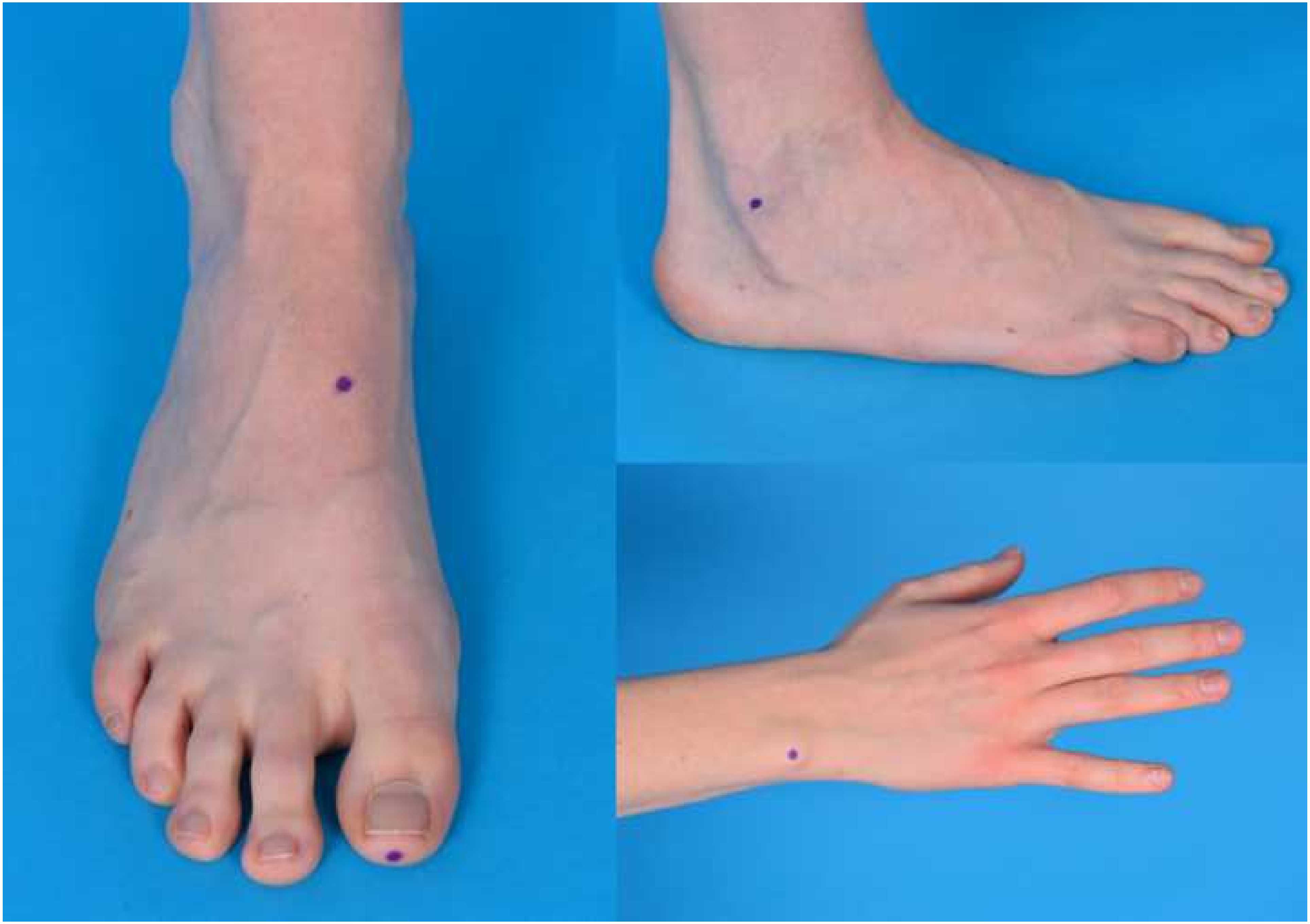

Figure 3

Click here to access/download;Figure;Fig 3.tif

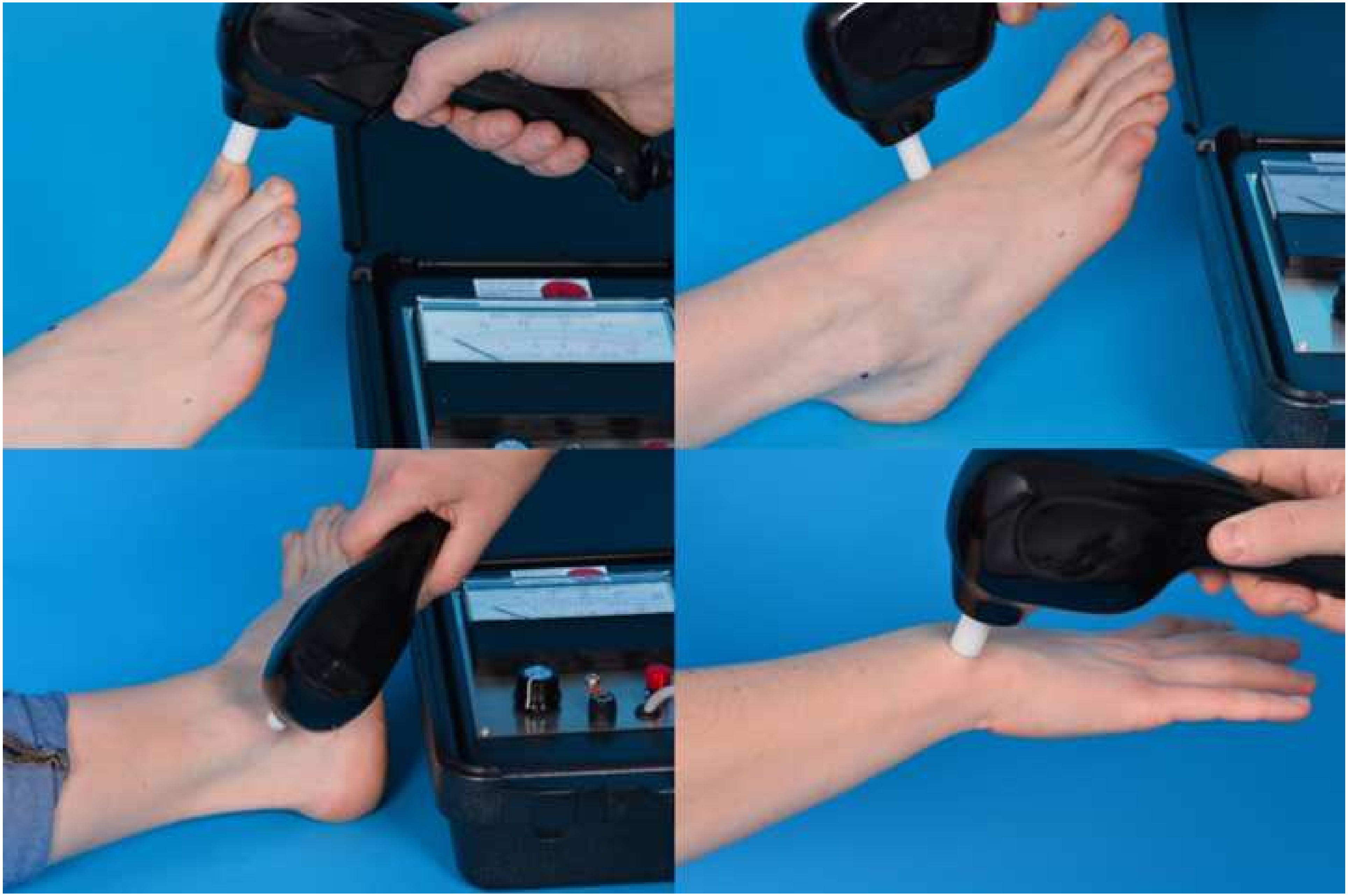

Figure 4 Click here to access/download;Figure;Fig 4.tif

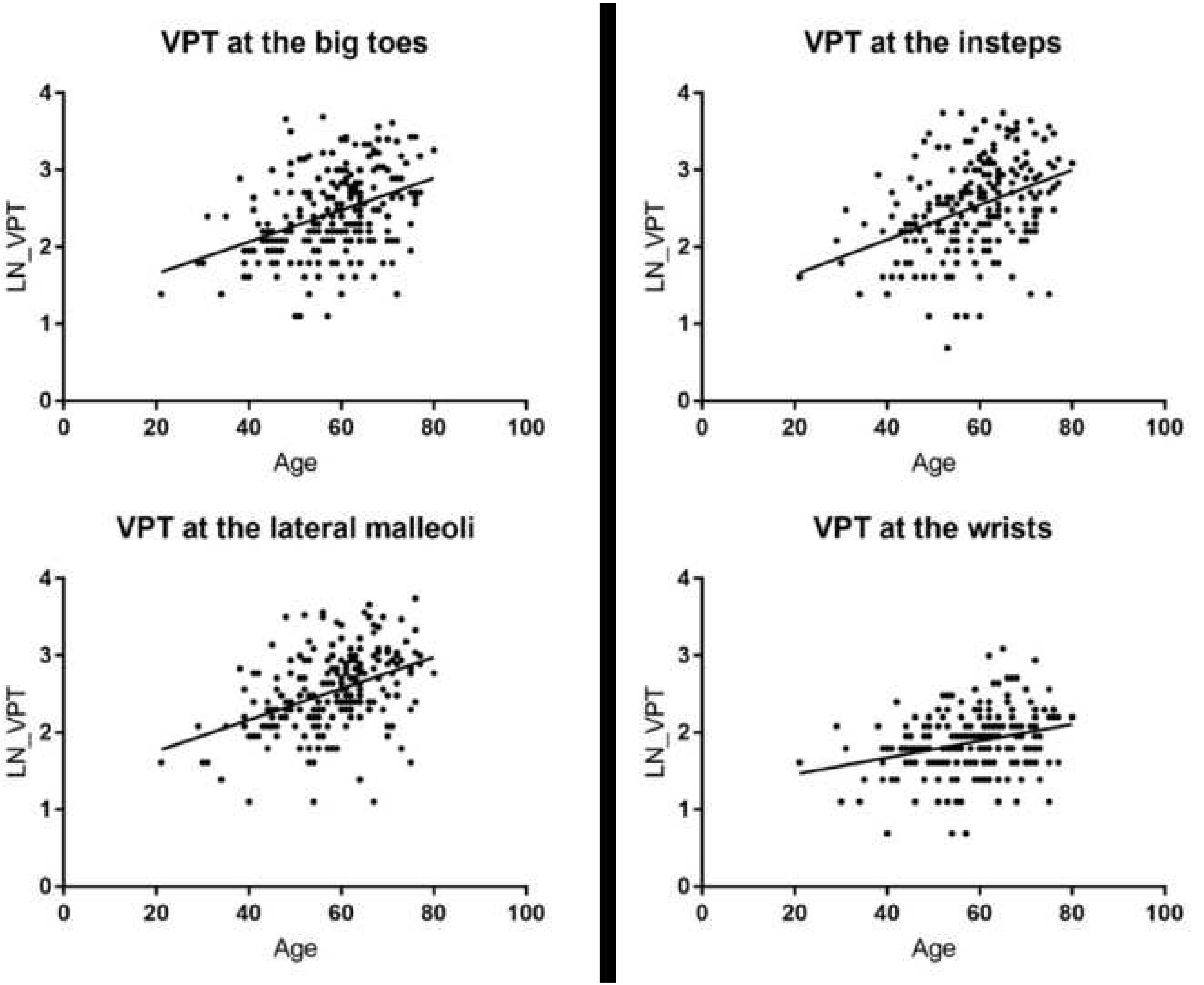

Figure 5

Click here to access/download;Figure;Fig 5.tif

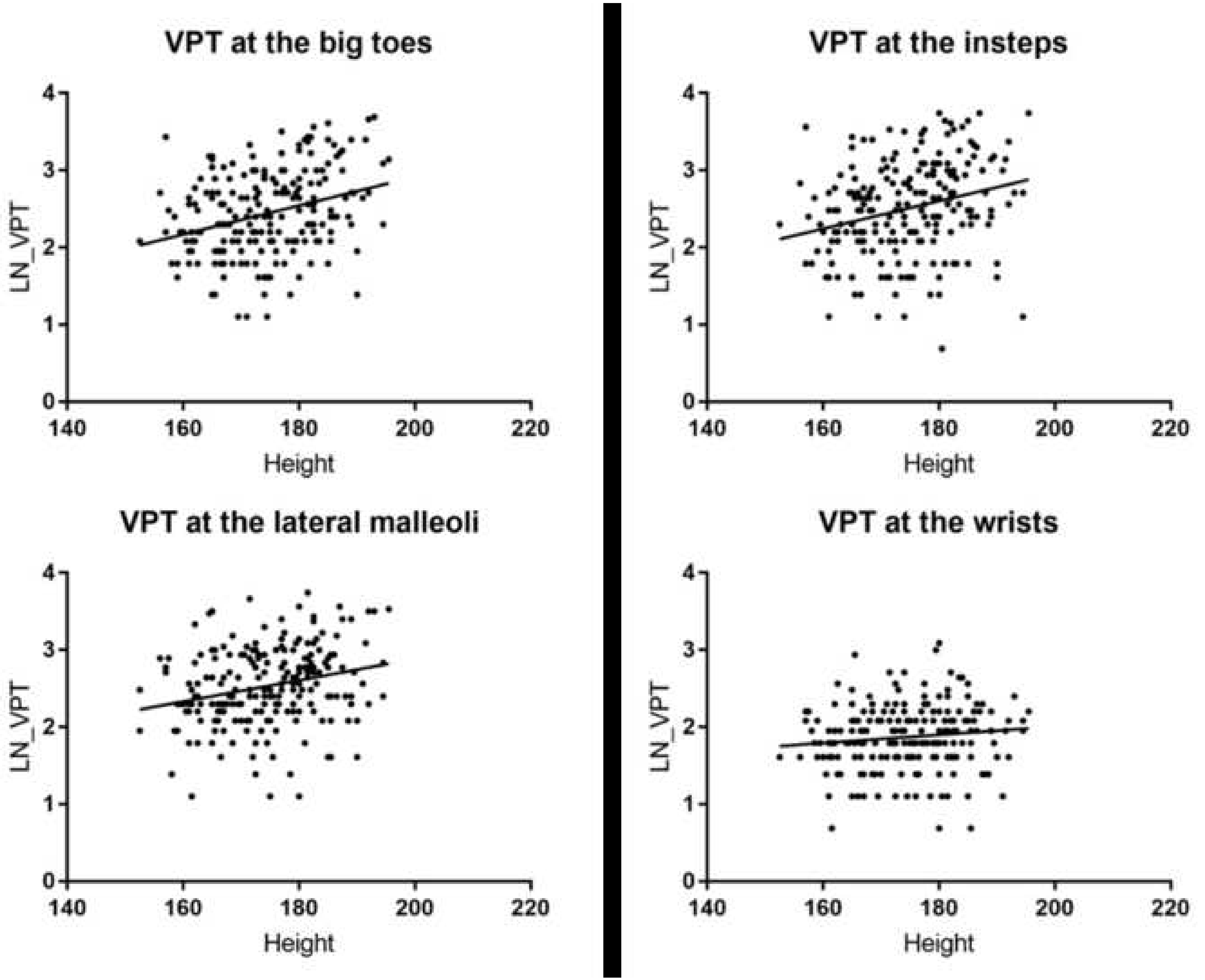

Figure 6

Click here to access/download;Figure;Fig 6.tif

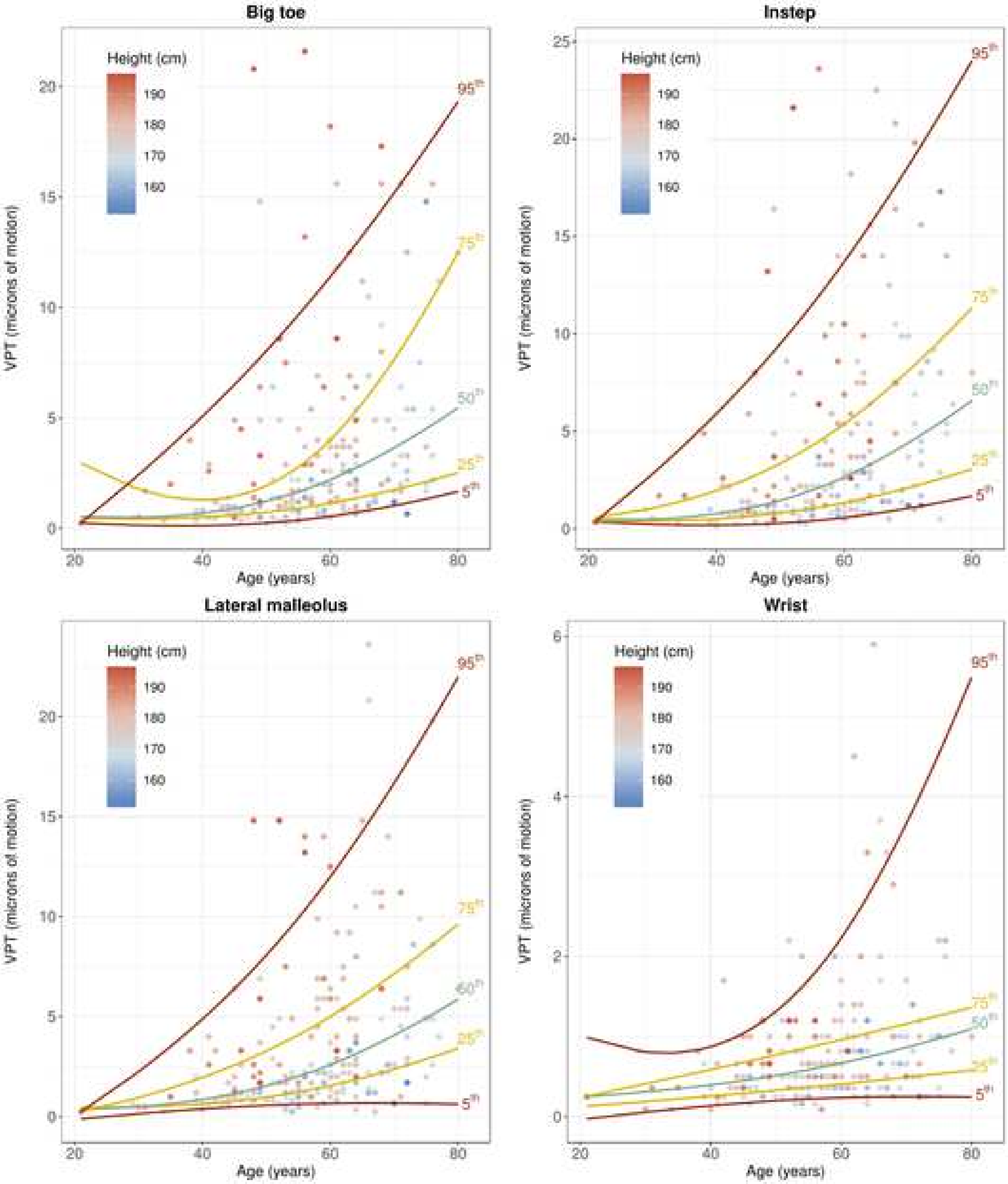

Figure 7

Click here to access/download;Figure;Fig 7.tif

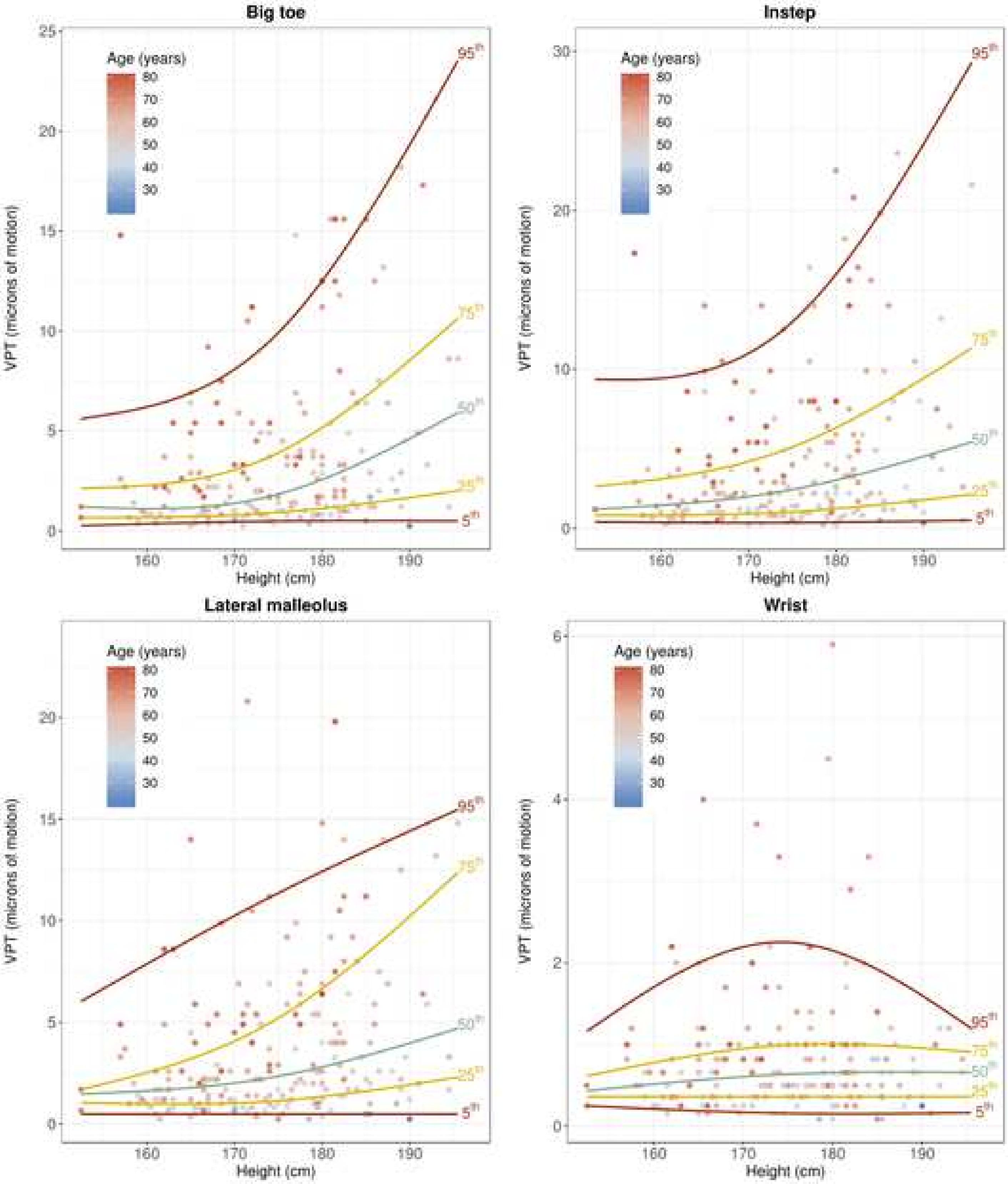

Supporting Information S1 Figure

Click here to access/download
**Supporting Information**
S1 Fig. Nomograms.pdf

**<u>Response to the reviewers' comments</u>**

**Reviewer #1**
We thank the reviewer for the comments on our manuscript; we have the impression that the manuscript clearly improved by implementing the suggestions.

<u>Comment 1:</u>
*Please shorten the introduction.*
<u>Response to comment 1:</u>
As you suggested we shortened the introduction by leaving out information on the qualitative 128-Hz tuning fork as this does not add vital background information for the current study. The current study focusses on determining vibration perception thresholds (VPT) and only a Rydell-Seiffer tuning fork and a biothesiometer are relevant devices that can measure VPT. Consequently, the introduction appears now more coherent.

<u>Comment 2:</u>
*Please highlight the practical implications.*
<u>Response to comment 2:</u>
We underlined the practical implications in the introduction by adding information on the need for normal values of VPT to follow patients that are treated for their large myelinated nerve fiber deficits (see manuscript lines 68-69). Moreover, we now address the clinical implications in the discussion, by mentioning the possible increase in prevalence of peripheral neuropathies due to population ageing (see manuscript lines 312-316). This underscores the importance of normal values for VPT to enable monitoring of these patients in an adequate and non-invasive way.

**Reviewer #2**
We thank the reviewer for the constructive comments that helped us to clarify a number of aspects of our manuscript.

<u>Comment 1:</u>
*The title of the paper is somewhat misleading since the Authors show that a large proportion of the subjects were not healthy although they did not have diabetes. My suggestion is to remove the word "healthy" from the title.*
<u>Response to comment 1:</u>
We agree with your comment and adjusted the title as suggested.

<u>Comment 2:</u>
*In the introduction the authors mention that the normal values have been presented previously by (at least) two groups (references 7 and 8). The paper by Bloom & al. was published already 1984 and examined vibration thresholds in 519 patients with varying ages from below 10 to up to 90 years! They do provide mean and range of vibration perception thresholds in different age groups at 3 different locations .Table II Bloom et. al.. The same is true for Maffei et. al. who unfortunately fail to describe which location was used. (I have contacted the authors but have not received any feedback ) Consequently, the claim that Bloom et. al. did not specify the normal values (page 3 line 69) is not true, and the current paper is the third one investigating the same questions. The novelty of this paper is that it gives the normal values in two locations not used previously (Instep and Wrist) and that the normal values are presented in microns, rather than Volts, which makes it difficult to compare with previous studies.*
<u>Response to comment 2:</u>
We agree that the explanation of the need for revisited normal values was unclear. It is correct that Bloom et al. already provided reference values for VPT. However, as already mentioned shortly by Bloom et al. and confirmed by other studies, height seems to be another important predictor to take into account next to age. Maffei et al. considered height as well as age but did not specify measurement locations. As a consequence, there are no optimal normal values for a clinical setting yet. In the current study, we provide normal values dependent on age and height; these normal values are of major importance in clinical practice, to follow up patients with large myelinated nerve

fiber deficits. In response to your comment, we added a more clear description of the lack of normal values based on age and height and the need for revised normal values to the introduction (see manuscript lines 63-69).

Comment 3:
*Introduction page 3 line 60-62: "By using the biothesiometer and thus detecting the first sensation of vibration, adaption to the sensation of vibration is less likely to develop." – This needs a reference.*
Response to comment 3:
Thank you for this suggestion. Accordingly, we added a reference for the argument that adaption to the sensation of vibration is less likely to develop when using a biothesiometer (see manuscript lines 56-57).

Comment 4:
*Page 3 line 62-63: "recent study has shown that a handheld biothesiometer is a superior tool to monitor changes in vibration sense" – should be: "superior to Rydel–Seiffer tuning fork" (the paper compares only these two methods).*
Response to comment 4:
We agree: a handheld biothesiometer is a superior tool compared to a Rydel-Seiffer tuning fork (see manuscript lines 57-60). We changed the sentence accordingly.

Comment 5:
*It seems that the authors had full access to medication history. Since 54.3 % of the patients used some medication and the mean age was high it would be interesting to know how many were using statins. This might influence the results (see e.g. Svendsen TK et al. Statin use and peripheral nerve function-A prospective follow-up study. Basic Clin Pharmacol Toxicol. 2019 Sep 10. 10.1111/bcpt.13320 )*
Response to comment 5:
Thank you for this suggestion. Accordingly, we added information on the use of statins (see manuscript Table 2). We also cited the suggested reference (reference 13 in the reference list) and an additional reference (reference 14 in the reference list). Because anti-retroviral medication is also well known for its adverse effects on peripheral nerve function, we have also added information on the use of this medication (none of the participants used such treatment) and an appropriate reference (reference 15 in the reference list).

Comment 6:
*How many patients did you have in each group? Our experience is that the variability increases dramatically after the age of 65.*
Response to comment 6:
Thank you for mentioning your observation. We included information on the number of subjects per age and height group in Table 7.
And indeed, the increase in variability can also be observed in the current study as well as an increase in the variability of VPT with increasing height (see manuscript Table 8).

Comment 7:
*As the authors mention the limitations of biothesiometer are reaction time of the subject and the ability to concentrate. Our experience measuring vibration perception thresholds with a different kind of device that can visualize the vibration perception thresholds is that approximately 5-10 % of the patients have difficulties in performing the measurement correct and these persons often report vibration thresholds that are too good, sometimes impossible to be true. My question is how many of the subjects had to do more than two measurements (deviation more that 20%) and why did you choose to use the lowest threshold? I would suggest that you try to exclude all the measurement where the subject seems to be guessing and calculate the mean between the two measurements if possible.*
Response to comment 7:
Considering the recording of the vibration perception, it remains a limitation of sensory testing that the measurements stay subjective and depend on the focus and reaction time of the subject (see manuscript lines 299-303). Therefore, to prevent other possible influencing factors, the study protocol of the current study was standardized (see manuscript lines 291-296): measurements were

performed in the same room, at similar room temperature and were repeated provided the first two measurements differed more than 20%. In response to your comment, we added information on the number of subjects that underwent a third measurement to Table 1. Furthermore, we changed the method and used the mean of all included measurements as you suggested (see manuscript lines 111-112). In the supporting information, we added a sensitivity analysis in which all subjects that underwent a third measurement were excluded and the mean between the two remaining measurements was calculated (see supporting information S2). Age and height were confirmed as independent determinants of VPT and the regression coefficients remained comparable to the ones calculated without excluding subjects that underwent a third measurement. Furthermore, we added a comparison of clinical characteristics between the group without a third measurement and the group that underwent a third measurement (see supporting information Table S2.2).

Comment 8:
*Although the regression equations give the mean values at different locations (or logarithm of the mean VPT?), more interesting and correct would be to present centile charts as Bloom et. Al. According to Bloom (and to our data) the variability of VPTs increases with increasing age and the confidence intervals should be presented for different age groups.*
Response to comment 8:
Thank you for this suggestion. Accordingly, we added Table 8 which presents the mean VPT and 95% confidence intervals for the different age and height groups.
The main difference compared to the study by Bloom et al. is that we also included height as a predictor of VPT because both height and age were independent predictors of VPT. Therefore, we added quantile charts of VPT as a function of age (see manuscript Figure 6) and height (see manuscript Figure 7). To include the respective second predictor in the chart, age and height were included as points with a color gradient. These colored points represent the participants included in the current study and are clustered around the quantiles which were calculated for the first predictor (age or height, respectively).
To be able to calculate normal values in clinical practice for every individual patient, we added nomograms in the supporting information which are based on the regression equations. These regression equations include the coefficients of the 95$^{th}$ percentile which was chosen as the cut-off for the normal value. By filling in the age and height of the patient on the outer scales of the nomograms and connecting the two points by drawing a straight line, it will be possible to read the normal value for the VPT for the respective patient from the middle scale.

Comment 9:
*It could also benefit if the data would be presented as medians+ confidence intervals for different age groups.*
Response to comment 9:
As suggested the data is now presented as mean VPT and 95% confidence interval for different age and height groups in Table 8.
