# Supplementary material for: Vibration threshold in non-diabetic subjects
Source: PLoS One. 2020 Oct 7;15(10):e0237733. doi: 10.1371/journal.pone.0237733 (PMC7540842; doi:10.1371/journal.pone.0237733)
Supplement: Attachment — Submitted filename: Response to reviewers.docx [file pone.0237733.s007.docx]

**Rebuttal letter**

**Reviewer #1**

Comment 1:

*Please shorten the introduction.*

Response to comment 1:

We thank the reviewer for this suggestion. Accordingly, we changed the introduction by shortening the detailed description of the Rydel-Seiffer tuning fork and the biothesiometer (see manuscript lines 49-56). This led to less repetition and in particular the introduction is shortened without losing coherence.

**Reviewer #2**

Comment 1:

*Data on tables 3-6 could be presented in one table. There are a lot of small tables also in the supplement part that could be presented together.*

Response to comment 1:

We thank the reviewer for this suggestion. Accordingly, we merged tables 3-6 in the main text and table S2.3-2.6 in the supplement part (see manuscript table 4 and S2.4).

Comment 2:

*Are the regression equations presented for logarithmic values? The authors state that “the obtained thresholds were log transformed”.*

Response to comment 2:

We thank the reviewer for this question. For the linear regression analyses, the obtained thresholds were log transformed to fulfill the assumption of normal distribution of the dependent variable. We used linear regression to identify clinical predictors of vibration perception thresholds. For the generation of the equations we used quantile regression, which does not require log transformation because quantile regression is a non-parametric statistical method (see following article: Furno M. Goodness of Fit and Misspecification in Quantile Regressions. J Educ Behav Stat. 2011;36(1):105-31). Therefore, the regression equations are not generated for logarithmic values but for the untransformed values that are directly obtained when measuring vibration sense with a biothesiometer. To clarify this we added a sentence stating that for generation of the regression equations the dependent variables were not log transformed (see manuscript lines 148-150).

Comment 3:

*Regarding last line in conclusion: (“The established equations can be used to determine loss of vibration perception in clinical practice.”) Is it possible to calculate standard deviations from these equations? That would be needed if one would use them to determine a specific threshold that is above normal. (Figures 6-7) could be used for that purpose.*

Response to comment 3:

We thank the reviewer for this suggestion. It should, however, be noted that due to the skewed distribution of the vibration perception threshold (VPT) data, we used quantile regression for these analyses. Since the quantile regression is non-parametric no standard deviations can be derived. The equations given in the manuscript correspond to the 95^th^ percentile resulting from the quantile regression analyses. The different conditional quantiles in Figures 6 and 7 are plotted using natural cubic splines of age and height, respectively. The 95^th^ percentile implies that all values measured above this border can be seen as above normal. To obtain standard deviations, a normal distribution of the VPT data would be required. We, however, found that it is not possible to transform the VPT data in such a way that a normal distribution of VPT data can be obtained. This is in agreement with earlier report by Bloom et al. (sample size: n=468 subjects) and Maffei et al. (sample size: n=150 subjects), who also found that it is difficult to transform VPT data in such a way that a normal distribution can be obtained (see manuscript reference 5 and 6). Unfortunately, it is therefore not possible to transform VPT data in such a way that standard deviations can be derived. Therefore, we used the 95^th^ percentile to provide a specific threshold that is above normal.

Comment 4:

*Line 57 “By using the biothesiometer and thus detecting the first sensation of vibration, adaption to the sensation of vibration is less likely to develop” According to reference 5, this is true for upper limbs but I can not see that this was the case for measurements in the lower limb.*

Response to comment 4:

We agree with the reviewer that it is unclear from reference 5 whether line 57 also applies to measurements in the lower limb. Marcuzzi et al. (reference 5) state in their manuscript that no significant systematic differences were found over the 3 consecutive measurements for vibration perception thresholds (VPT) at any site using the vibrameter. The described testing sites in this study were in the upper limb the distal interphalangeal joint of the index finger and the ulnar styloid process, and in the lower limb the proximal metatarsal joint of the great toe and the medial malleolus. They conclude in their discussion that the learning or habituation effect applies for measuring vibration disappearance thresholds (VDT) but not VPT. In their discussion, they use an unclear sentence in which they mention that a small but systematic decrease in vibration threshold was detected in the lower limb between repeat applications. However, they do not specify whether this applies also for VPT or only VDT. Accordingly, we changed the particular sentence in the manuscript, now excluding the lower limbs from the statement (see manuscript lines 56-58).

**Reviewer #3**

Comment 1:

*Please make note of the published study by Rinkel et al. on normative data, with decline in sensibility, of static, moving and one-point discrimination.*

Response to comment 1:

We thank the reviewer for this comment. The results of the study by Rinkel et al. are indeed very interesting and do strenghten the current findings. It was particulary interesting that spatial discrimination was also found to deteriorate with ongoing age. Furthermore, vibration sense and spatial discrimination were shown to negatively correlate in a population of diabetic subjects. The negative correlation can be explained by the scale of a Rydel-Seiffer tuning fork when determining vibration perception: the highest score 8 reflects a low vibration amplitude and the lowest score 0 a high vibration amplitude. Therefore, Rinkel et al. concluded that with a decrease of spatial discrimination vibration sense also decreases in the respective subject. Accordingly, we added a statement to the discussion in which we mention the concordance in finding a decline of both vibration sense and spatial discrimination with ongoing age and the correlation of both sensory tests (see manuscript lines 269-272).

Comment 2:

*Please make a statement on comparability or superiority of VPT measurements measured with the Biothesiometer compared to Rydel Seiffer tuning fork.*

Response to comment 2:

In the introduction section of the manuscript we made the following statements on the comparability and superiority of VPT measurements measured with the biothesiometers in comparison to the Rydel-Seiffer tuning fork: “To examine vibration sense in a quantitative way vibration perception thresholds (VPTs) can be determined either by using a Rydel-Seiffer graduated tuning fork or a biothesiometer [2-4]. The VPT determined using the Rydel-Seiffer tuning fork is defined as the value nearest to the point where vibration is no longer perceived which is contrary to a biothesiometer which measures the VPT as the point at which the first sensation of the vibration appears [2,4]. It has been shown for the upper limbs that by using the biothesiometer and thus detecting the first sensation of vibration, adaption to the sensation of vibration is less likely to develop [3]. Moreover, a recent study has shown that a handheld biothesiometer is a superior tool compared to the Rydel-Seiffer graduated tuning fork to monitor changes in vibration sense over time, which makes it ideal for long-term follow-ups [3]. It is also noteworthy that a biothesiometer has a low inter-rater variability [5], particularly important in clinical practice and in large studies in which several investigators perform the measurements.” (see manuscript lines 49-63).

Comment 3:

*Introduction: Please provide values on low inter-rater variability of a biothesiometer.*

Response to comment 3:

We thank the reviewer for this comment. Bloom et al. did not mention the precise values of the low-inter-rater variability of a biothesiometer. Nevertheless, we added data on the inter-rater variability of the biothesiometer with an intra-class correlation coefficient and provided the values found in this study in the results section (see manuscript lines 241-245).

Comment 4:

*Methods: Where exactly was the vibrator button applied on big toe? Plantarly or dorsally?*
Response to comment 4:

In response to this comment, we added a more precise description on the location where the vibrator button was applied which was the tip of the big toe (see manuscript lines 105-107).

Comment 5:

*Table 1: display it as percentages. How many patients needed a third assessment (32/205 for example). Actually, what do these numbers say? Did they feel less also? Where they positioned in the upper centiles of the data?*

Response to comment 5:

We agree with the reviewer that displaying the numbers as percentages is more informative. Accordingly, we changed displaying of the numbers to percentages (see manuscript Table 1). The participants that underwent a third measurement did not cluster around the upper centiles, meaning they did not present with higher vibration perception thresholds, but on the contrary had very different vibration perception thresholds. Therefore, our explanation for the need of a third measurement is that these participants had problems with focusing to identify the perception threshold. Quantative sensory testing remains a subjective report mainly dependent on a subjects’ focus and participants sometimes named that identifying the precise point when they start to perceive the vibration is an unfamiliar task (see manuscript lines 292-296).

Comment 6:

*Statistical analysis: why not add hba1c, vit b12 as predictors?*

Response to comment 6:

We did not include HbA1c and vitamin B12 as predictors as they both are associated to peripheral neuropathies. HbA1c was part of the diabetes mellitus definition used in the current study. Therefore, we excluded subjects with vitamin B12 deficiency and diabetic subjects as we were aiming for a healthy population to determine normal values (see manuscript lines 78-80). Because this limits variability, and therefore statistical power for finding associations, we did not include HbA1c or vitamin B12 as predictors. Furthermore, we were not aiming to identify predictors for abnormal vibration perception as in peripheral neuropathy but for normal values of vibration perception. We are currently working on a study examining possible predictors of polyneuropathy in which we are also including HbA1c and vitamin B12 in the prediction model. This study will not have the intention of describing normal values, but rather of finding potential predictors of polyneuropathy, for which the largest possible variation of each variable in the population will be exploited.

Comment 7:

*Statistical analysis: F to enter and to remove is not usually described in this way.*

Response to comment 7:

We thank the reviewer for noticing this. Accordingly, we changed the sentence and gave the P value that was used to include a variable in the model (see manuscript lines 143-144).

Comment 8:

*Statistical analysis: could you specify how many raters were involved in this part of the study (inter-rater reliability)? Were all 26 assessors involved? How many repeated measurements did they do?*

*Response to comment 8:*

We thank the reviewer for these questions. To asses the inter-rater reliability of the biothesiometer, 25 different investigators measured the vibration perception threshold in 41 different subjects repeatedly. Each subject underwent three measurements, each performed by a different investigator. For the measurements the described protocol was followed. We added more information on this to the methods section (see manuscript line 120).

Comment 9:

*Table 2: Please, specify which parameters were normal and non-normal, i.e., average ±SD for parametric and median (IQR) for non-parameteric variables.*

Response to comment 9:

Accordingly, we included information on the distribution of the included variables (see footnote Table 2).

Comment 10:

*Determinants of the VPT: please provide unadjusted and adjusted values of predictors (that is: results from univariable and multivariable analyses)*

Response to comment 10:

To accomodate the comment of the reviewer, we added the results of the univariable unadjusted linear regression analyses to the revised version of the manuscript (see manuscript Tables 3 and S2.3).

**Reviewer #4**

Comment 1:

*Sample size is too small. How did you come up with this sample size?*

Response to comment 1:

We based the sample size on a manuscript in which the minimal number of subjects required for reference interval studies by using non-parametric statistics was mentioned (see following article: Jones G, Barker A. Reference Intervals. Clin Biochem Rev. 2008;29(Suppl 1):S93-S97). In this manuscript it was calculated that a number of 119 subjects is sufficient to determine the 90% confidence intervals of the 2.5^th^ and 97.5^th^ percentile of a population. As we agree with the reviewer that this intuitively is a relatively low number, we decided to increase the number of included subjects to at least 200. To accommodate the comment of the reviewer, we added these sample size considerations, together with the reference to the methods section of the revised version of the manuscript (see mansucript lines 167-169).

Comment 2:

*Values of VPT are equipment specific and machines are not standardized. It is a common observation that the values form 2 different machines are not the same. Can the results be generalized to other equipments?*

Response to comment 2:

We thank the reviewer for this question. The equipment used in this study is regularly calibrated by the medical technical department of the University Medical Center Groningen according to the manufacturers’ recommendations. Therefore, when using a biothesiometer (Bio-thesiometer®; Bio Medical Instrument Co, Ohio, USA) that vibrates at a frequency of 120 Hz with an amplitude range of 0.01-25.5 μm the equations for normal values can be generalized. Accordingly, we added a statement on the calibration of the machine used for this study to the method section (see manuscript lines 99-100).

Comment 3:

*Mean value of VPT is less than 2, whereas for diabetic neuropathy thresholds such as 15 and 25 are used. Given the low mean value, minor differences after adustment for age and height are practically non-significant and unikely to be benificial in any clinical condition. In addition VPT values are partly subjective with significant variation and minor differences are unlikely to be of any importance. Which clinical condition do you think your research is going to be beneficial?*

Response to comment 3:

We thank the reviewer for this question. Unfortunately, we are not aware of the thresholds you are naming for diabetic neuropathy. We are carefully assuming that you are referring to thresholds of 15 and 25 Volt. In our manuscript we are using the unit microns of motion which is the unit referred to by the company producing the biothesiometer (see manuscript lines 114-117). When converting the value you are giving in your comment, 2.0 microns of motion equals 14 Volt. We will add the converting table to the supplementary information (S3 Fig.). To address your question on which clinical condition can benefit from this study we would like to point out that mainly diseases affecting large myelinated fibers are of interest. This is because when assessing vibration sensation the large myelinated fiber function is tested. Within this group, polyneuropathy is a common affliction which knows different causes and understandably the causes affecting the large myelinated fibers are of main interest.

Comment 4:

*Median age of the study population of 59 years is somewhat strange to generate normative values and adjustments for age/height. You shoud have chosen a healthy populaion below 50 years of age.*

Response to comment 4:

We thank the reviewer for this suggestion. As we mentioned in the manuscript, this is indeed one of our limitations which may lead to an underestimation of the threshold for subjects younger than 39 years old (see manuscript lines 296-302). Loss of vibration sensation can be seen as part of sensory testing when diagnosing polyneuropathy. A study of Visser et al. which was carried out in the Netherlands has shown that the incidence of all polyneuropathies increases from the age of 40 years onwards and has their highest incidence in the age group of 75 to 79 years (see following article: Visser NA, Notermans NC, Linssen RSN, van den Berg LH, Vrancken AFJE. Incidence of Polyneuropathy in Utrecht, the Netherlands. Neurology. 2015;84(3):259-64). Therefore, normal values for vibration perception which is one part of the sensory testing when diagnosing polyneuropathy are in particular needed for the elderly. Accordingly, we added a statement on this to the discussion section (see manuscript lines 302-307). Lastly, we would like to point out that the normal values given in this study are adjusted for age and height. Therefore, we simultaneously corrected for the age of the study population.

Comment 5:

*VPT is not as sensitive a test for diabetic neuropathy compared to nerve conduction studies. Can you enumeate which clinical condions is VPT preferred as diagnostic test for early detection.*

Response to comment 5:

We thank the reviewer for this comment. Nerve conduction studies are considered as the golden standard to assess diseases affecting large myelinated nerve fibers (see following article: Graham Gwathmey K, Pearson KT. Diagnosis and Management of Sensory Polyneuropathy. BMJ. 2019;365:l1108). In clinical practice sensory testing appears to be a less invasive alternative method in comparison to nerve conduction studies. In order to be able to to draw a valid conclusion on a subjects’ peripheral neuropathy several different sensory tests are needed among which vibration testing is essential. An example to implement this in clinical practice is the modified Toronto Clinical Neuropathy Score which was designed to capture signs and symptoms of diabetic sensory and sensorimotor polyneuropathy. It includes next to patient reported symptom scores five different sensory tests: testing of pain sensation, temperature sensation, light touch, position sense, and vibration sensation (see following article: Bril V, Tomioka S, Buchanan RA, Perkins BA, mTCNS Study Group. Reliability and Validity of the Modified Toronto Clinical Neuropathy Score in Diabetic Sensorimotor Polyneuropathy. Diabet Med. 2009;26(3):240-6). Examination of vibration sense with the biothesiometer determines the vibration perception threshold of a subject. It is one of the few sensory tests that gives a quantified result. In this study we provided normal values for vibration perception thresholds which can be used to quantify the loss of vibration sense in large myelinated neuropathies which makes it ideal for the follow-up of a patient (see manuscript lines 310-322).

Comment 6:

*There are too many figures and tables which need to be cut-down to 1-2 each.*

Response to comment 6:

We agree with the reviewer that numbers of figures and tables can be cut down. Accordingly, we merged tables 3-6, 7-8 in the main text and tables S2.3-2.6, S2.7-2.8 in the supplement part (see manuscript table 4 and 5, and S2.4 and S2.5).
