# Supplementary material for: Vibration threshold in non-diabetic subjects
Source: PLoS One. 2020 Oct 7;15(10):e0237733. doi: 10.1371/journal.pone.0237733 (PMC7540842; doi:10.1371/journal.pone.0237733)
Supplement: Attachment — Submitted filename: Response to reviewers.docx [file pone.0237733.s008.docx]

**Rebuttal letter**

**Reviewer #3**

Comment 1:

*It is wise to state that the Rydel-Seiffer tuning fork with weights attached tests at 64 Hz. Without weights, one tests at 128 Hz.*

Response to comment 1:

We thank the reviewer for this comment. Accordingly, we added the statement on the Rydel-Seiffer tuning fork to the Introduction section in which we compared the testing procedure of a Rydel-Seiffer tuning fork to the one of a biothesiometer (see manuscript lines 51-52).
